# The influence of chirality on the behavior of oligonucleotides inside cells: revealing the potent cytotoxicity of G-rich l-RNA†

**DOI:** 10.1039/d2sc05511b

**Published:** 2022-12-30

**Authors:** Chen-Hsu Yu, Jonathan T. Sczepanski

**Affiliations:** a Department of Chemistry, Texas A&M University College Station Texas 77843 USA jon.sczepanski@chem.tamu.edu

## Abstract

Due to their intrinsic nuclease resistance, mirror image l-oligonucleotides are being increasingly employed in the development of biomedical research tools and therapeutics. Yet, the influence of chirality on the behavior of oligonucleotides in living systems, and specifically, the extent to which l-oligonucleotides interact with endogenous biomacromolecules and the resulting consequences remain unknown. In this study, we characterized the intracellular behavior of l-oligonucleotides for the first time, revealing important chirality-dependent effects on oligonucleotide cytotoxicity. We show that exogenously delivered l-oligonucleotides have the potential to be highly cytotoxic, which is dependent on backbone chemistry, sequence, and structure. Notably, for the sequences tested, we found that single-stranded G-rich l-RNAs are more cytotoxic than their d-DNA/RNA counterparts, exhibiting low nanomolar EC_50_ values. Importantly, RNA-seq analysis of differentially expressed genes suggests that G-rich l-RNAs stimulate an innate immune response and pro-inflammatory cytokine production. These data not only challenge the general perception that mirror image l-oligonucleotides are nontoxic and nonimmunogenic, but also reveal previously unrecognized therapeutic opportunities. Moreover, by establishing sequence/structure toxicity relationships, this work will guide how future l-oligonucleotide-based biotechnologies are designed and applied.

## Abstract 2

We determined the influence of chirality on the behavior of oligonucleotides inside human cells, revealing that mirror-image l-oligonucleotides have the potential to be highly cytotoxic.

## Graphical abstract

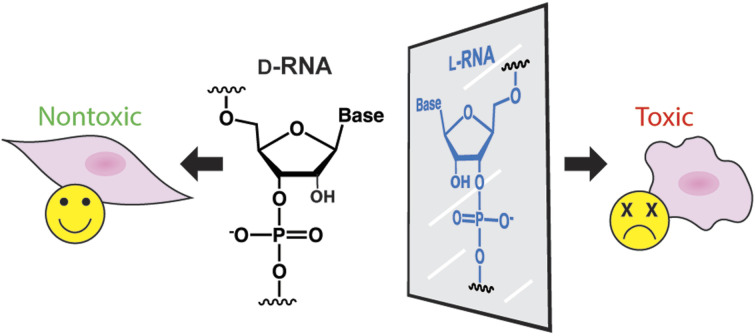

## Introduction

**Fig. 1:**
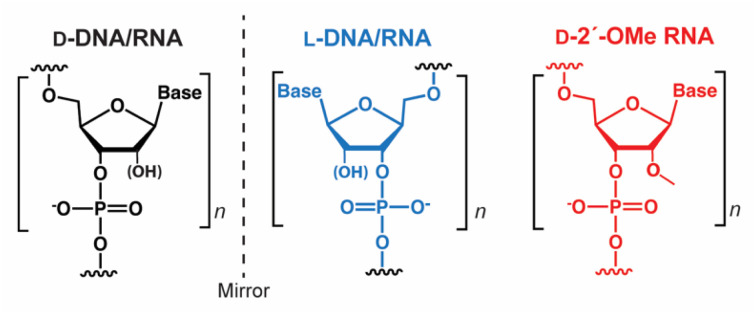
l-Oligonucleotides are the synthetic enantiomer of native d-oligonucleotides.

Native nucleic acids, d-DNA and d-RNA, are chiral molecules as a result of their d-(deoxy)ribose sugars. As such, they have enantiomers, referred to as mirror image l-DNA and l-RNA, which consist of l-(deoxy)ribose sugars (Fig. 1). Although l-oligonucleotides no longer exist in nature, they can be prepared synthetically in the laboratory, where they have been studied since the 1970s.^1^ From a biotechnology perspective, l-oligonucleotides have several advantageous properties relative to their native counterparts.^1^ Most notably, l-oligonucleotides are highly resistant to degradation by cellular nucleases, providing them with superior stability in harsh biological environments.^2,3^l-Oligonucleotides are also expected to avoid potentially toxic off-target interactions with endogenous nucleic acids because d- and l-oligonucleotides are incapable of forming contiguous WC base pairs with each other.^2,4^ Furthermore, as enantiomers, d- and l-oligonucleotides have the same physical properties in terms of duplex thermostability and hybridization kinetics.^4–6^ Thus, well-established design principles can be directly applied to l-oligonucleotides without further optimization, representing a key advantage over other chemically modified oligonucleotides.^7,8^ These favorable characteristics have fuelled the recent development of many promising l-oligonucleotide-based biotechnologies, including l-aptamers,^9–12^ microarrays,^2^ molecular sensors,^13^ live cell imaging probes,^14–16^ and drug delivery.^17,18^

Given the stereospecific nature of biomolecular interactions, the behavior of d- and l-oligonucleotides within living systems is expected to be different. For example, the inability of d- and l-oligonucleotides to form contiguous WC base pairs with each other implies that l-oligonucleotides will interact far less with endogenous nucleic acids relative to their native counterparts.^2,4^ However, l-oligonucleotides are still susceptible to non-specific protein interactions, which are likely to be distinct from their native counterparts. Nevertheless, the influence of chirality on the behavior of oligonucleotides in living systems and, specifically, the extent to which l-oligonucleotides interact with endogenous biomacromolecules has not been studied carefully. Given the large number of proteins that have been shown to bind nucleic acids in a nonspecific or “promiscuous” manner, it is reasonable to predict that interactions across the mirror are abundant, potentially leading to undesired effects. Indeed, we recently showed that l-RNA binds tightly to the polycomb repressive complex 2 (PRC2) and prevents it from interacting with its intended targets.^19^ If l-oligonucleotides are to be routinely employed in living cells and organisms, then it is imperative that we understand how they interact with these environments and their potential consequences. Such studies may also reveal whether chirality as a design parameter can be harnessed to induce and/or modulate a desired biological response, opening the door to new applications. In this study, we conducted the first detailed characterization of how l-oligonucleotides interact with biological systems at the cellular level, revealing important chirality-dependent behaviors of l-oligonucleotides. Notably, we show that G-rich l-oligonucleotides have potent cytotoxicity and a dramatic impact on gene expression, suggesting they make extensive interactions with endogenous proteins.

## Results and discussion

### G-rich l-RNA is cytotoxic to human cells

**Fig. 2:**
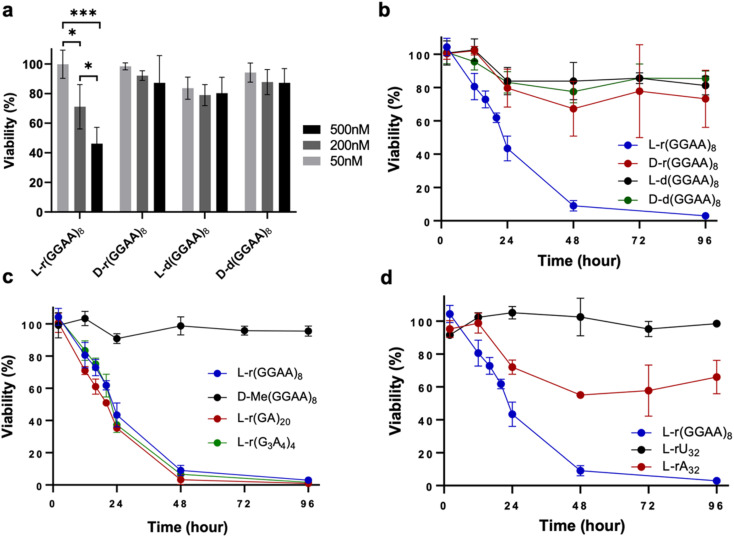
G-rich l-RNA is uniquely cytotoxic. (a) Viability of HeLa cells 24 hours post-transfection with the indicated oligonucleotide as measured by CCK-8 assay. Data are mean ± S.D. (*n* = 3 biological replicates). *P < 0.05; **P < 0.01; ***P < 0.001. (b–d) Time-dependent viability assay (CCK-8) of HeLa cells treated with 200 nM of the indicated oligonucleotide. Data are mean ± S.D. (*n* = 3 biological replicates).

We previously reported that guanine (G)-rich l-RNAs, but not other sequences, bound tightly to PRC2 *in vitro*, suggesting that G-rich sequences have a propensity for interacting with native proteins. Therefore, we chose the model G-rich sequence (GGAA)_8_ as a starting point for our studies. We prepared both d and l stereoisomers of (GGAA)_8_ DNA and RNA (d/l-d(GGAA)_8_ and d/l-r(GGAA)_8_, respectively) (Table S1†). As an initial test for disruption of cellular pathways, we transfected HeLa cells with the different (GGAA)_8_ variants and measured cell viability after 24 hours using an CCK-8 assay. Unless stated otherwise, all oligonucleotides were transfected into cells using Lipofectamine 3000 (see ESI†). Of the four (GGAA)_8_ variants tested, only l-r(GGAA)_8_ exhibited a strong concentration-dependent decrease in cell viability after 24 hours, suggesting that the l-RNA strand was uniquely cytotoxic upon transfection (Fig. 2a). We confirmed these results by conducting a time-dependent toxicity assay over the course of 96 hours (Fig. 2b). Notably, while cells treated with d-r(GGAA)_8_ showed no cytotoxic effects during this timeframe, l-r(GGAA)_8_ killed nearly all cells after just 48 hours. The half-maximal effective concentration (EC_50_) of l-r(GGAA)_8_ was determined to be 58 ± 26 nM (Table 1 and Fig. S1†), which is ∼5-fold lower than d-r(GGAA)_8_. We extracted the various (GGAA)_8_ oligonucleotides from cells after 24 hours and analysed their length by gel electrophoresis. The results revealed that comparable levels of full-length material for all four versions of (GGAA)_8_ were present inside cells after 24 hours (Fig. S2†). The persistence of d-r(GGAA)_8_ and d-d(GGAA)_8_ is likely due to the Cy5 dye positioned at the 3′-end, which has been shown to greatly increase the half-life of oligonucleotides in serum-supplemented media and inside cells by protecting against 3′-exonuclease degradation.^20^ Importantly, the presence of full-length d-d(GGAA)_8_ and d-r(GGAA)_8_ inside cells after 24 hours indicated that their much lower cytotoxicity relative to l-r(GGAA)_8_ cannot be attributed to nuclease degradation. Unsurprisingly,^21^ the 2′-*O*-methyl (2′-OMe) RNA version of d-r(GGAA)_8_, d-Me(GGAA)_8_, showed no visible signs of cytotoxicity in HeLa cells (Fig. 2c and Table 1), highlighting the divergent effects of 2′-OMe and l-RNA modifications on cytotoxicity. Two other G-rich l-RNAs, l-r(GA)_20_ and l-r(G_3_A_4_)_4_, showed a similar toxicity profile as l-r(GGAA)_8_ (Fig. 2c) and even lower EC_50_ values (Table 1 and Fig. S1†), indicating that potent cytotoxicity is a common characteristic of G-rich l-RNA. Consistent with the (GGAA)_8_ series, the d-RNA versions of these RNAs, d-r(GA)_20_ and d-r(G_3_A_4_)_4_, were at least 30-fold less cytotoxic than their l-RNA counterparts (Table 1 and Fig. S1†). In addition, we found that all three G-rich l-RNAs l-r(GGAA)_8_, l-r(GA)_20_ and l-r(G_3_A_4_)_4_ were cytotoxic to MCF7 cells (Fig. S3†), demonstrating that the observed cytotoxicity is not specific to HeLa cells.

**Table 1:** Half maximal effective concentrations (EC_50_) for the different oligonucleotides in HeLa cells (48 hour treatment)

Sequence	EC_50_ (nM)
l-r(GGAA)_8_	58 ± 26
d-r(GGAA)_8_	279 ± 68
l-d(GGAA)_8_	2056 ± 854
d-d(GGAA)_8_	634 ± 253
d-Me(GGAA)_8_	1974 ± 687
l-r(GC/GC)	>3000
l-r(G_3_A_4_)_4_	12 ± 3
d-r(G_3_A_4_)_4_	339 ± 122
l-r(GAAA)_10_	42 ± 14
l-rA_32_	191 ± 42
l-r(GA)_20_	28 ± 12
d-r(GA)_20_	>3000
l-r(GA)_15_	34 ± 15
l-r(GA)_10_	55 ± 24
l-r(GA)_5_	413 ± 197
l-r(GU)_20_	133 ± 60

To determine whether the observed cytotoxicity of l-RNA was selective for G-rich sequences, we also examined l-poly[A] (l-rA_32_) and l-poly[U] (l-rU_32_) (Fig. 2d). HeLa cells treated with l-rU_32_ were mostly viable (>90%) after 96 hours, whereas cells treated with l-rA_32_ were ∼60% viable after the same time. The EC_50_ for l-rA_32_ was calculated to be 191 ± 42 nM, indicating that it was still much less cytotoxic than those containing both G and A (EC_50_ ∼ 10–50 nM) (Table 1 and Fig. S1†). Nevertheless, the observed cytotoxicity of l-rA_32_ suggested that there may be an effect from purine-rich sequences in addition to G-rich sequences on the cytotoxicity of l-RNA. To test this, we prepared an l-RNA containing only G and U, l-r(GU)_20,_ and found that it was ∼4-fold less toxic than the analogous sequence containing only G and A, l-r(GA)_20_ (Table 1 and Fig. S1†). Thus, in addition to guanine, the overall purine content appears to contribute the cytotoxicity of G-rich l-RNA. One possible explanation is that the greater hydrophobicity of A relative to the other nucleobases^22^ results in increased cytotoxic l-RNA–protein interactions.^23^ Additional studies are needed to explore this idea further. Finally, we attempted to address whether cytotoxicity was proportional to the percent G in a sequence by comparing l-r(GAAA)_10_ (25% G) and l-r(GGGA)_10_ (75% G) to l-r(GGAA)_8_ and l-r(GA)_20_, both of which contain 50% G. Unfortunately, l-r(GGGA)_10_ formed aggregates under almost all conditions tested, which precluded cellular delivery and analysis. The EC_50_ of l-r(GAAA)_10_ was found to be within error of both l-r(GGAA)_8_ and l-r(GA)_20_, despite having half the number of Gs relative to As (Table 1 and Fig. S1†). Thus, while we cannot comment on sequences with >50% G, these data suggest that for G/A-rich l-RNA sequence with 25–50% G, the cytotoxicity is not proportional to the percent G.

Overall, these results demonstrate that G-rich l-RNAs have the potential to be highly cytotoxic to human cells, especially in the presence of adenine (*i.e.*, G/A-rich sequences). Cytotoxic and antiproliferative activities have previously been reported for G-rich d-oligonucleotides.^24–28^ These results now indicate that such properties extend across the chiral mirror. These results also show that the stereochemistry of an oligonucleotide can influence its cytotoxicity and, in the case of G-rich sequences, we find that l-RNA is considerably more cytotoxic than its d-counterparts for those sequences tested herein. To the best of our knowledge, the first time any cytotoxicity has been assigned to l-oligonucleotides.

### Cytotoxicity of G-rich l-RNAs is dependent on structure and length

The cytotoxicity of oligonucleotides has been shown to be dependent on secondary structure and folding. For example, a positive correlation between G-quadruplex (G4) formation and cytotoxicity has been reported for G-rich d-DNA.^28^ In the case of G-rich l-RNA, potent cytotoxicity was observed for both G4-forming sequences (*e.g.*, l-r(GGAA)_8_) and those that cannot fold into G4s (*e.g.*, l-r(GA)_20_) (Fig. 2 and Table 1),^19^ suggesting that the cytotoxic effects are mostly independent of the potential for G4 formation. In support of this notion, we obtained circular dichroism spectra of two cytotoxic sequences l-r(GGAA)_8_ and l-r(GA)_20_ under conditions used during transfection (25 mM Tris pH 7.4, 1 mM EDTA, and 50 mM KCl in Opti-MEM), as well as under intracellular K^+^ concentrations (140 mM KCl) (Fig. S4†). These data confirmed that l-r(GGAA)_8_, but not l-r(GA)_20_, form folded G4 structures under both sets of conditions. Thus, while we can't completely rule out the involvement of G4 structures, they do not appear to be the major determinant of l-RNA toxicity.

**Fig. 3:**
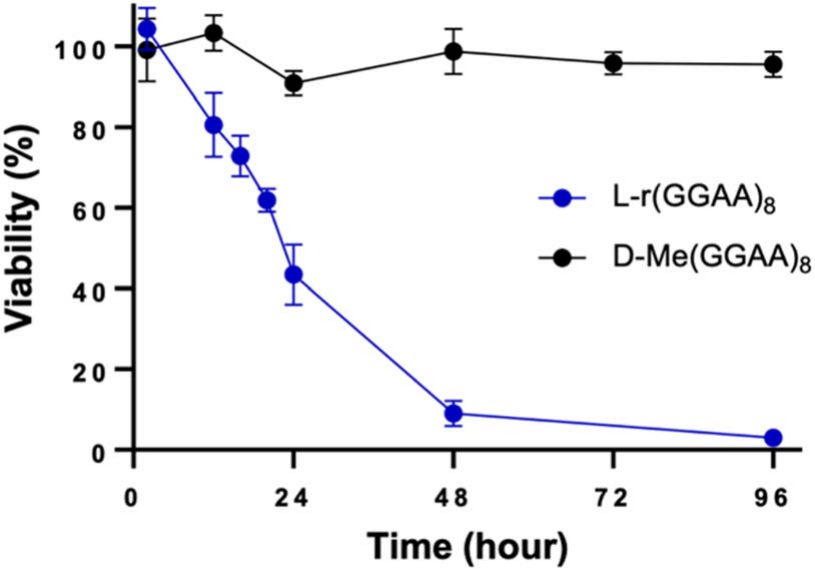
Time-dependent viability assay (CCK-8) of HeLa cells treated with 200 nM of l-r(GC/GC) hairpin. l-r(GGAA)_8_ is shown for reference. Data are mean ± S.D. (*n* = 3 biological replicates).

In addition to G4s, single-stranded (ss) and double-stranded (ds) oligonucleotides can also influence cytotoxic interactions.^29^ To test this, we prepared a G-rich l-RNA, l-r(GC/GC), that contained the same number of Gs as l-(GGAA)_8_ but was designed to fold into a hairpin such that the majority of G residues where base paired. Cells treated with l-r(GC/GC) hairpin (200 nM) showed no cytotoxic effects after 96 hours (Fig. 3). The EC_50_ for l-r(GC/GC) hairpin was calculated to be >3 μM (Table 1 and Fig. S1†). Thus, we conclude that the cytotoxicity of G-rich l-RNA is dependent on the RNA being single-stranded.

In addition to structure, we also examined the influence of length on cytotoxicity of l-RNA. We prepared a series of incrementally shorter versions of l-(GA)_20_ (Table S1†). As shown in Table 1, l-r(GA)_20_, l-r(GA)_15_, l-r(GA)_10_, which contain 20, 15, and 10 Gs, respectively, were equally cytotoxic to HeLa cells (EC_50_ ∼ 30–50 nM) following transfection. However, when the length was further reduced to contain only five Gs (l-r(GA)_5_), the cytotoxicity was reduced dramatically (EC_50_ = 413 ± 197 nM). This suggests that a minimum of ten closely spaced Gs is sufficient for l-RNA cytotoxicity.

### Cellular uptake and intracellular distribution of l-oligonucleotides

**Fig. 4:**
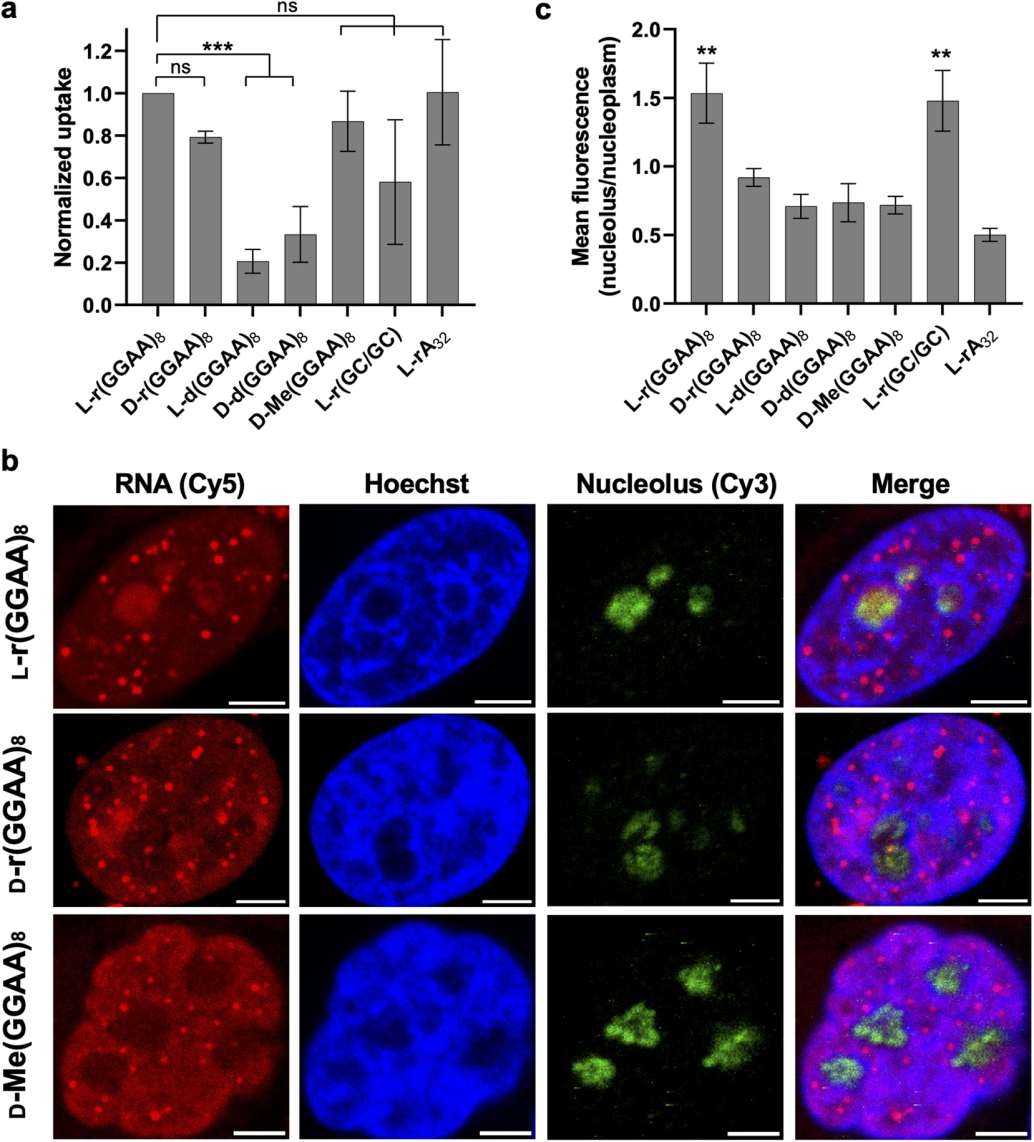
Uptake and localization of l-oligonucleotides into HeLa cells. (a) Cellular uptake of the various oligonucleotides studied in this work. HeLa cells were transfected with 200 nM oligonucleotide and uptake was measured 2 hours later by flow cytometry. Data are mean ± S.D. (*n* = 3 biological replicates). ***P < 0.001. (b) Representative fluorescent confocal microscopy images of HeLa cells transfected with 200 nM of the indicated oligonucleotides. See also Fig. S5.† Nucleolus was stained with an anti-fibrillarin antibody and Cy3-labeled secondary antibody. All scale bars: 5 μm. (c) Nucleolar enrichment of G-rich l-RNA. Data is the mean fluorescent intensity within the nucleolus divided by the mean fluorescent intensity within the nucleoplasm. Values greater than one indicate nucleolar enrichment (*n* = 4 cells). **P < 0.01.

We next examined cellular uptake and localization of l-oligonucleotides with the goal determined whether these factors were correlated with cytotoxicity. We first measured the uptake of the various 3′-Cy5-labeled oligonucleotides (200 nM) into HeLa cells by flow cytometry two hours after transfection (Fig. 4a). In general, the RNA series of oligonucleotides were uptaken by HeLa cells better than the DNA series (d/l-d(GGAA)_8_) when transfected by Lipofectamine 3000. However, the difference in uptake between DNA and RNA series was only about 2–3-fold, whereas the EC_50_ values for the d/l-d(GGAA)_8_ were at least 10-fold higher than any of the cytotoxic l-RNA strands. Among the RNA series, little correlation was found between uptake and cytotoxicity. All d-RNA and l-RNA oligonucleotides tested, as well as d-Me(GGAA)_8_, had similar uptake into HeLa cells, despite l-r(GGAA)_8_ being substantially more cytotoxic than the others (Fig. 4a and Table 1). Overall, while we can't completely rule out a role for uptake, these data suggest that the potent cytotoxicity of G-rich l-RNA relative to the other oligonucleotides tested herein is not due to increased cellular uptake.

Fluorescent confocal microscopy was then used to determine the subcellular localization of the different 3′-Cy5-labeled oligonucleotides post transfection (Fig. 4b and S5†). After 2 hours all oligonucleotides primarily localized into the cell nucleus, consistent with earlier reports showing that synthetic oligonucleotides transfected with lipid-based reagents rapidly accumulate in the nucleus.^21,30,31^ However, there were interesting differences in the subnuclear localization of these strands. l-r(GGAA)_8_, d-r(GGAA)_8_, and d-Me(GGAA)_8_ formed discrete nuclear foci that were not present in cells treated with other oligonucleotides (Fig. 4b and S5†). Similar structures have been widely observed upon transfection of backbone-modified antisense oligonucleotides (ASOs) into cells, and have been attributed to non-specific protein interactions, and in particular, with nuclear paraspeckle-associated proteins (*e.g.*, P54nrb).^21,23,29,32^ The lack of nuclear foci for d/l-d(GGAA)_8_ and l-r(CG/GC) hairpin are consistent with this model, as DNA and double-stranded oligonucleotides do not interact with paraspeckle proteins.^29^ While future studies are needed to determine the exact nature and composition of the foci formed by these RNAs, these observations suggest that l-r(GGAA)_8_ can seed the formation of protein-rich nuclear structures when transfected into cells. Another major difference among the oligonucleotides examined was nucleolar localization. Whereas most of the strands were excluded from the nucleolus, l-r(GGAA)_8_ was significantly enriched in this compartment (Fig. 4c and S5†). l-r(GGAA)_8_ was also the only oligonucleotide to both form nuclear foci and accumulate in the nucleolus, suggesting that the combination of these activities underly its potent cytotoxic effects. This notion is consistent with previous studies showing that ASO-mediated sequestration of nuclear proteins, including paraspeckle proteins, into the nucleolus induces cellular toxicity through nucleolar stress.^21,29^

Together, these data show that G-rich l-RNA form nuclear foci and accumulate in the nucleolus when transfected into HeLa cells, behaviors that are highly indicative of cytotoxic protein interactions. These data also demonstrate that in addition to sequence and structure, the subcellular localization and trafficking of oligonucleotides is dependent on its chirality.

### G-rich l-RNAs induce apoptosis

**Fig. 5:**
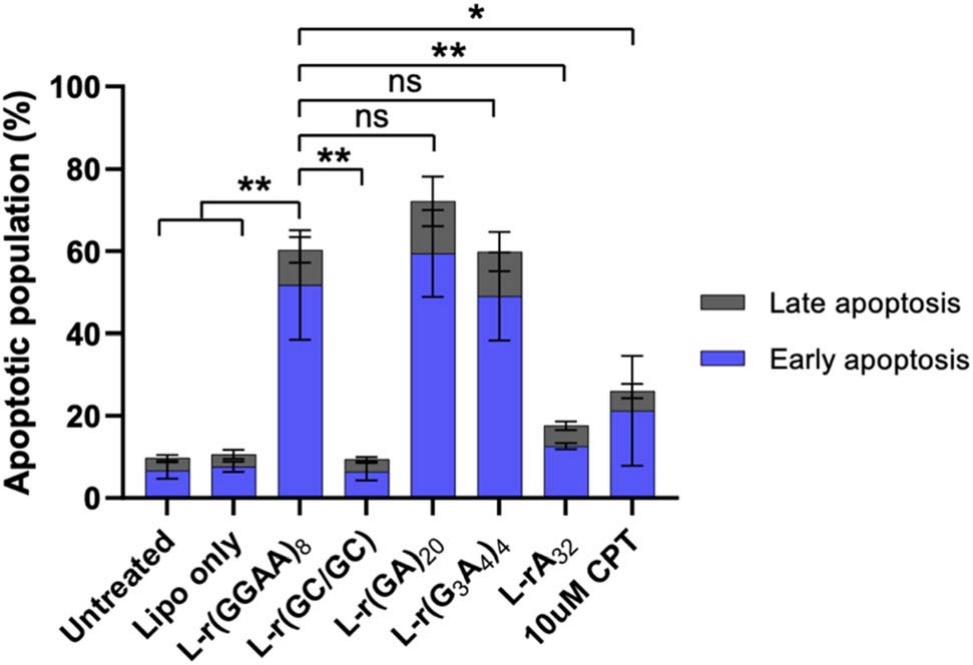
G-rich l-RNA induces apoptosis. HeLa cells were transfected with 200 nM of the indicated oligonucleotide and the apoptotic population was determined after 24 hours. Sum of early (annexin V positive; blue) and late (annexin V and PI positive; grey) apoptotic cells are shown. Data are mean ± S.D. (*n* = 3 biological replicates). *P < 0.05; **P < 0.01.

To determine the type of cell death related to cytotoxic G-rich l-RNA, HeLa cells were stained with annexin V and propidium iodide (PI) 24 hours post transfection and fluorescence was measured by flow cytometry. AnnexinV detects the externalization of phosphatidylserine on early apoptotic cells, while PI is used to detect necrotic or late apoptotic cell due to the loss of membrane integrity.^33^ Very few (<10%) apoptotic or necrotic cells were detected in untreated and Lipofectamine-only treated cells after 24 hours (Fig. 5 and S6†). However, treatment with cytotoxic G-rich l-RNAs resulted in ∼50% apoptotic cells (annexin V positive and PI negative) and 10–20% late apoptotic cells (annexin V and PI positive) following 24 hour exposure. The extent of apoptotic cells for G-rich l-RNAs exceeded that of camptothecin (CPT), a common positive control for apoptosis. None of the toxic l-RNAs induced significant necrosis (annexin V negative and PI positive) (Fig. S6†). As expected, the non-toxic l-RNA hairpin l-r(GC/GC) did not trigger apoptosis or necrosis. l-rA_32_ also induced far less apoptosis than any of G-rich l-RNAs, consistent with its lower overall cytotoxicity. Together, these results suggest that toxic G-rich l-RNA induce apoptotic cell death.

### Differential gene expression induced by G-rich l-RNA indicate activation of innate immunity

**Fig. 6:**
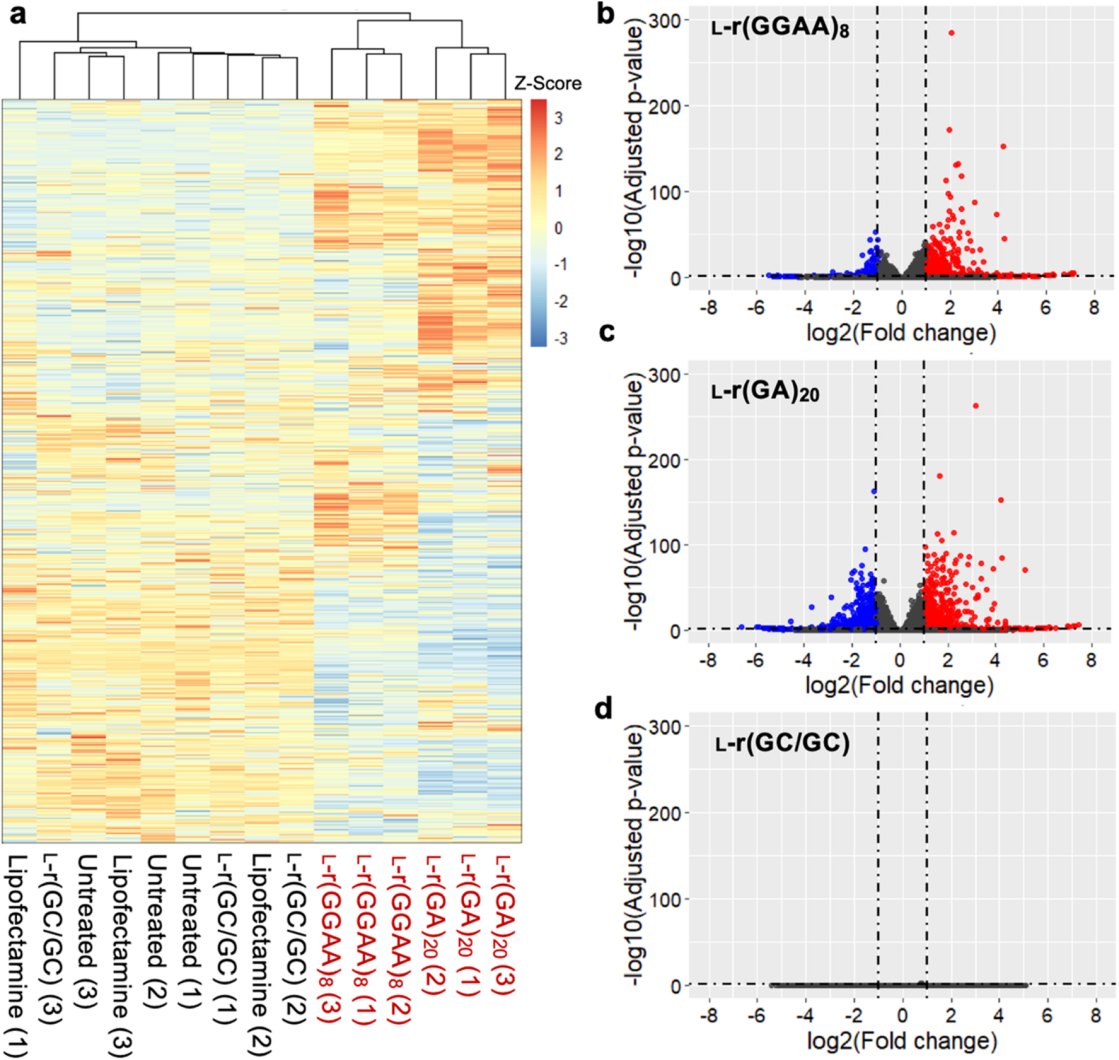
Differentially expressed genes induced by l-r(GGAA)_8_ and l-(GA)_20_. (a) Unsupervised hierarchical clustering analysis (Euclidean distance) of gene expression in HeLa cells treated with G-rich l-RNAs compared to untreated cells or Lipofectamine only controls (*n* = 3 biological replicates). Numbers in parenthesis indicates biological replicate number. (b–d) Volcano plots of differential expressed genes (relative to Lipofectamine only control) in HeLa cells treated with (b) l-r(GGAA)_8_, (c) l-r(GA)_20_, (d) l-r(GC/GC). Genes with log_2_ fold change > 1 are colored red; genes with log_2_ fold change < −1 are colored blue (*P* < 0.05 for all).

To better understand the source of cytotoxicity for G-rich l-RNAs, and to reveal the extent of their biological interactions, we performed RNA-seq analysis of total RNA isolated from HeLa cells 12 hours after treatment with 200 nM l-r(GGAA)_8_ and l-r(GA)_20_. Cells treated with Lipofectamine only and non-toxic l-r(GC/GC) served as controls for these experiments. As shown in Fig. 6a, gene expression differed significantly in cells treated with cytotoxic l-r(GGAA)_8_ and l-r(GA)_20_ compared to the controls, which were clustered together. Interestingly, gene expression profiles for l-r(GGAA)_8_ and l-r(GA)_20_ were also clustered separately, indicating that they interact differently with cells. While l-r(GGAA)_8_ is capable of folding into a G-quadruplex structure, l-r(GA)_20_ is not (Fig. S4†), representing a potential source for these differences. Treatment with l-r(GGAA)_8_ caused significant upregulation of 507 genes (log_2_(ratio) ≥ 1, *P*-value < 0.05) and downregulation of 267 genes (log_2_(ratio) ≤ −1, *P*-value < 0.05) relative to the Lipofectamine only control (Fig. 6b and ESI File 1†). In cells treated with l-r(GA)_20,_ 689 genes were upregulated and 1015 genes were downregulated relative to the Lipofectamine only control (Fig. 6c and ESI File 1†). The expression level of several significantly dysregulated genes was confirmed by qRT-PCR, which showed good agreement with the RNA-seq data (Fig. S7†). No genes were differentially expressed in cells treated with non-toxic l-r(GC/GC). Overall, these data reveal that G-rich l-RNA induce dramatic perturbations in gene expression levels, suggesting extensive engagement with cellular proteins.

**Fig. 7:**
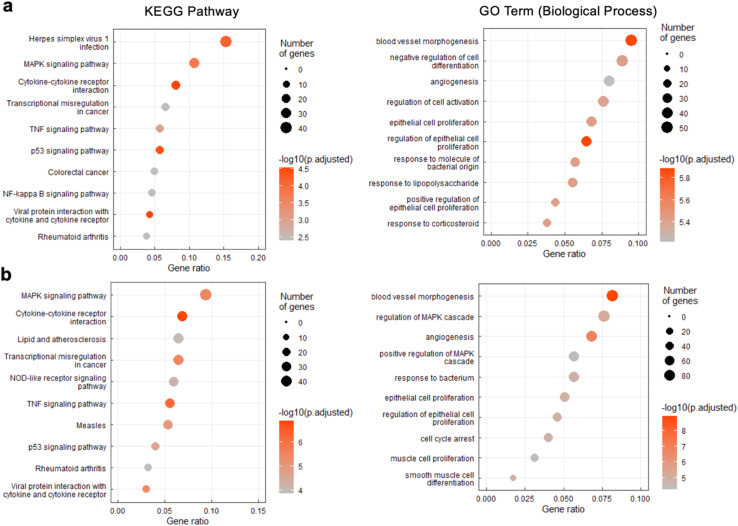
KEGG pathway and Gene Ontology (GO) enrichment analysis of differentially expressed genes following treatment of HeLa cells with 200 nM of either l-r(GGAA)_8_ (a) or l-r(GA)_20_ (b). The top-10 enriched KEGG pathways (left) and GO terms (biological process) (right) are listed for each RNA.

To interpret the potential biological consequence of changes in the transcriptome caused by l-r(GGAA)_8_ and l-r(GA)_20_, over-representation analyses were performed to identify biological pathways and processes enriched in differentially expressed genes. Although there were some differences, KEGG pathway analysis revealed a common set of enriched pathways for l-r(GGAA)_8_ and l-r(GA)_20_, including mitogen-activated protein kinase (MAPK) signaling pathway, cytokine–cytokine receptor interaction, (tumour necrosis factor) TNF signaling pathway, p53 signaling pathway, and transcriptional misregulation (Fig. 7). Collectively, these pathways strongly suggest that l-r(GGAA)_8_ and l-r(GA)_20_ stimulate an innate immune response, possibly *via* engagement of pattern-recognition receptors such as Toll-like receptors (TLRs).^34–36^ Consistently, several key pro-inflammatory cytokines central to the innate immune response, including TNF, interleukin (IL)-1, IL-6, IL-12, and CXCL8, were among the most highly overexpressed genes (Table S2†). Moreover, the gene ontology (GO) biological process analyses identified blood vessel morphogenesis, angiogenesis, and cell proliferation among the top 10 enriched terms for both cytotoxic l-RNAs (Fig. 7), which is consistent with cytokine overexpression, and in particular, the very high level (>100-fold) of the angiogenic chemokine CXCL1 (Table S2†).^37–39^ A number of pro-apoptotic genes associated with inflammatory cytokine production and downstream p53 activation were also upregulated in response to l-r(GGAA)_8_ and l-r(GA)_20_ (Table S2†).^40,41^ In support of TLR receptor involvement, siRNA-mediated knockdown of TLR3 and/or TLR7 resulted in a ∼70% reduction in TNF expression following G-rich l-RNA treatment as compared to WT cells (Fig. S8†). While TLR engagement and immune system-related cytotoxicity has been documented for synthetic oligonucleotides,^42–45^ including those with unnatural sugars,^46^ strong immunostimulatory actives by G-rich l-RNA was surprising given the complete stereochemical inversion of the sugar backbone, as well as prior reports concluding that l-oligonucleotides have very low immunogenic potential.^9,47^ These prior studies, however, did not examine G-rich l-RNAs, highlighting the importance of sequence activity relationship studies. We acknowledge that immune system-related cytotoxicity may not be the only cause of apoptosis induced by G-rich l-RNA. For example, accumulation of gapmer ASOs in the nucleoli, which we also observed for l-r(GGAA)_8_, was shown to induce p53 activation and apoptotic cell death.^21^ Further experiments beyond the scope of this work will be required to fully identify the pathways involved in l-RNA toxicity, as well as the specific receptors (*e.g.*, TLRs) responsible for these signalling outcomes.

## Conclusions

In conclusion, we characterized the intracellular behavior of l-oligonucleotides for the first time, revealing that the chirality of an oligonucleotide can have a profound effect on its behavior in living systems. Most notably, we found that single-stranded G-rich l-RNAs were highly cytotoxic to human cells, whereas d-RNA versions of the same sequence showed mild or no cytotoxicity. While the number of sequences examined herein is likely too limited to generalize that G-rich l-RNAs are more cytotoxic that their d-counterparts, our results highlight the profound influence of stereochemistry on the behavior of oligonucleotides inside cells. Furthermore, we showed that cytotoxic l-RNA induce dramatic perturbations in gene expression levels. Given that l-oligonucleotides are incapable of forming contiguous WC base pairing interactions with native DNA and RNA, we propose that these effects are the results of l-RNA–protein interactions, such has been observed previously for cytotoxic d-oligonucleotides. While future proteomics studies will be needed for confirmation and to identify key l-RNA–protein interactions responsible for the observed cytotoxic effects, an analysis of differentially expressed genes suggests that G-rich l-RNA stimulate an innate immune response and pro-inflammatory cytokine production. Thus, these data challenge the general perception that mirror image l-oligonucleotides are nontoxic and nonimmunogenic. Moreover, this work demonstrates that the antiproliferative properties of G-rich d-oligonucleotides (*e.g.*, AS1411), which are currently being pursued as anticancer agents, extends across the chiral mirror to G-rich l-RNA.^23–27,48^

Due to their favorable properties, such as nuclease resistance, l-ON are being increasingly employed in biomedical applications. However, our results emphasize that caution should be taken when employing l-oligonucleotides in cells and organisms. Moving forward, it will be important to better characterize off-target interactions underlying these cytotoxic effects and to establish detailed structure toxicity relationships. Such information could be used to better predict toxicity from sequence and potentially allow for toxic motifs to be eliminated at the design stage, which provided the key motivation for this work. Towards this goal, this study offers some preliminary design considerations for minimizing the potential toxic effects of l-oligonucleotides. For example, based on the sequences tested herein, l-DNA appears to be less cytotoxic than l-RNA, making it a better choice for most biomedical applications. However, if l-RNA must be used, long (>10 nt), single-stranded regions rich in G residues should be avoided, especially in the context of G/A-rich sequences. Furthermore, complementary interactions (*i.e.*, hybridization) appear to mitigate the cytotoxic effects of G-rich l-RNA, as evident by l-r(GC/GC) hairpin. This suggests that the cytotoxicity and potential antiproliferative activities of l-RNA could be regulated through programmable base pairing interactions, opening the door to more-targeted anticancer therapies. Finally, while most applications of l-oligonucleotides may seek to evade an immune response (*e.g.*, Spiegelmers), l-oligonucleotides could also be highly useful as immunostimulants (*e.g.*, vaccine adjuvants), especially considering their lack of off-target hybridization and superior nuclease resistance. Thus, further evaluation of the immunostimulatory effects of l-RNA, as well as the specific pathways and receptors involved, is highly warranted.

Overall, this work represents an important step in understanding the intracellular behavior of l-oligonucleotides and the establishment of an l-oligonucleotide “interactome”, both of which are expected to have a broad impact on how future l-oligonucleotide-based technologies are designed and applied. Our results also reveal previously unrecognized therapeutic opportunities for l-oligonucleotides. More broadly speaking, this work should motivate similar investigations into other modified oligonucleotides, such as XNAs, that are also perceived to be highly bioorthogonal, which may reveal unforeseen biological effects.

## Data availability

Raw RNA-seq read and processed data are available in the NCBI GEO repository under the accession number GSE205338. The complete list of differentially expressed genes for l-r(GGAA)_8_ and l-r(GA)_20_ can be accessed in the ESI File 1 document.† All other data generated during all experiments is available from the author upon reasonable request.

## Author contributions

Chen-Hsu Yu: methodology, investigation, formal analysis, visualization, writing – review & editing. Jonathan Sczepanski: conceptualization, writing – original draft, supervision, project administration, funding acquisition.

## Conflicts of interest

The authors declare no competing financial interests.

## Supplementary Material

SC-014-D2SC05511B-s001

SC-014-D2SC05511B-s002
