# Correction: The influence of chirality on the behavior of oligonucleotides inside cells: revealing the potent cytotoxicity of G-rich l-RNA

**DOI:** 10.1039/d3sc90119j

**Published:** 2023-06-23

**Authors:** Chen-Hsu Yu, Jonathan T. Sczepanski

**Affiliations:** a Department of Chemistry, Texas A&M University College Station Texas 77843 USA jon.sczepanski@chem.tamu.edu

## Abstract

Correction for ‘The influence of chirality on the behavior of oligonucleotides inside cells: revealing the potent cytotoxicity of G-rich l-RNA’ by Chen-Hsu Yu *et al.*, *Chem. Sci.*, 2023, **14**, 1145–1154, https://doi.org/10.1039/D2SC05511B.

The originally published version of this manuscript contained an incorrect figure for Fig. 3. The original Fig. 3 showed a toxicity time-course for d-Me(GGAA)_8_ compared to l-r(GGAA)_8_ when the intended graphic should have shown a toxicity time-course for hairpin l-r(GC/GC) compared to l-r(GGAA)_8_. The correct version of Fig. 3 is as follows, and replaces that within the original manuscript.

**Fig. 1:**
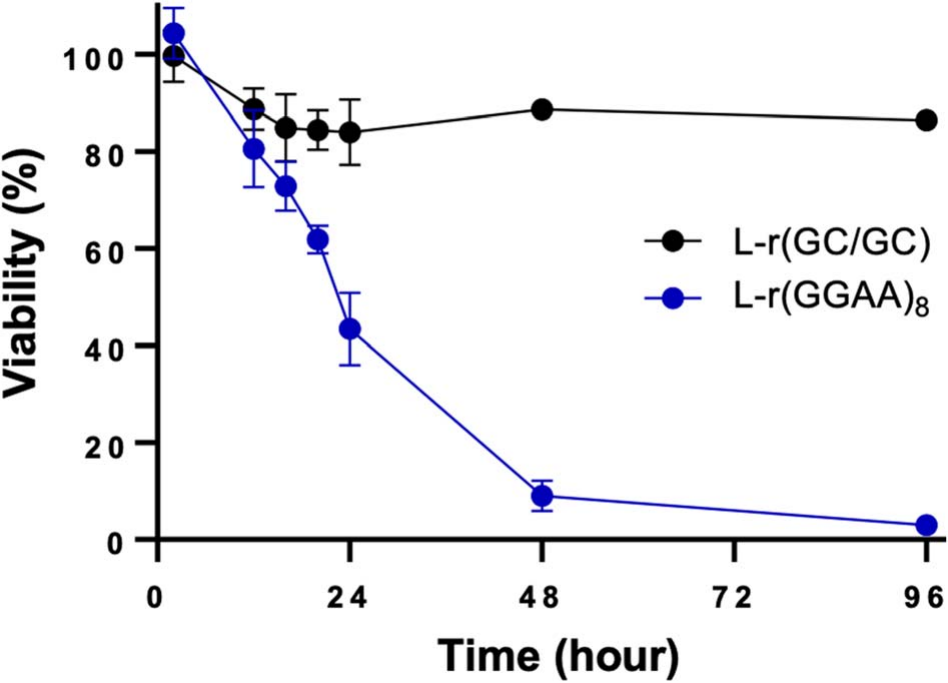
Time-dependent viability assay (CCK-8) of HeLa cells treated with 200 nM of l-r(GC/GC) hairpin. l-r(GGAA)_8_ is shown for reference. Data are mean ± S.D. (*n* = 3 biological replicates).

The Royal Society of Chemistry apologises for these errors and any consequent inconvenience to authors and readers.

## Supplementary Material

