# Supplementary material for: The influence of chirality on the behavior of oligonucleotides inside cells: revealing the potent cytotoxicity of G-rich l-RNA
Source: Chem Sci. 2022 Dec 30;14(5):1145–54. doi: 10.1039/d2sc05511b (PMC9891384; doi:10.1039/d2sc05511b)
Supplement: SC-014-D2SC05511B-s002 [file SC-014-D2SC05511B-s002.pdf]

Electronic Supplementary Material (ESI) for Chemical Science.
This journal is © The Royal Society of Chemistry 2022

Supporting Information

The Influence of Chirality on the Behavior of Oligonucleotides Inside Cells: Revealing the Potent Cytotoxicity of G-Rich L-RNA

Chen-Hsu Yu[1] and Jonathan T. Sczepanski[1,*]

[1]Department of Chemistry, Texas A&M University, College Station, Texas 77843

S1. Supplementary Text.

Materials and Methods.

General. Oligonucleotides were prepared by solid-phase synthesis on an Expedite 8909 DNA/RNA Synthesizer using manufacturer recommended protocols. D-Nucleoside phosphoramidites and solid supports, as well as all synthesis reagents were purchased from Glen Research (Sterling, Va). L-Nucleoside phosphoramidites and solid supports were purchased from ChemGenes (Wilmington, Ma). Lipofectamine 3000 and cell culture media (DMEM, OptiMEM) were purchased from ThermoFisher, (Waltham, MA). Sulfo-Cy5 N-hydroxysuccinimide (NHS) ester was purchased from Lumiprobe Corp. (Hunt Valley, MD). Apoptosis assay reagent including AnnexinV-FITC and camptothecin were purchased from Abcam (Cambridge, United Kingdom). Purelink RNA mini kit was purchased from Invitrogen (San Diego, CA). All other reagents were purchased from Sigma Aldrich (St. Louis, MO).

Oligonucleotide purification and labeling. Prior to use, all oligonucleotides were purified by 20% denaturing polyacrylamide gel electrophoresis (PAGE; 19:1 acrylamide:bisacrylamide). Purified oligonucleotides were excised from the gel and eluted overnight at 23 °C in a buffer consisting of 200 mM NaCl, 10 mM EDTA, and 10 mM Tris (pH 7.6). The solution was then filtered to remove gel fragments, and eluted oligonucleotides were concentrated using an Amicon Ultra Centrifugal Filter (MilliporeSigma, Burlington, MA) having a membrane pore size of 3 kDa. Following concentration, all samples were desalted by ethanol precipitation, and the concentration determined by absorbance at 260 nm on a NanoDrop 2000c (ThermoFisher, Waltham, MA). The identity of all oligonucleotides was confirmed using a Thermo Scientific Q Exactive Focus ESI mass spectrometer (Figure S9 – S23).

Sulfo-Cy5 N-hydroxysuccinimide (NHS) ester (Lumiprobe Corp., Hunt Valley, MD) was conjugated to the 3' end of oligonucleotides via a 3' amino modification installed at the time of synthesis. Conjugation reactions were performed by combining the amino modified oligonucleotide (100 µM) with the dye NHS ester (5 mM final concentration) in 0.1 M sodium bicarbonate buffer (pH 8.5). The reaction was vortexed intermittently over a 16-hour period at 23 °C. Samples were then ethanol precipitated and the labeled oligonucleotide was purified by 20% denaturing PAGE as mentioned above. Oligonucleotide stock solution was prepared by diluting the purified oligonucleotide to 10 µM in folding buffer (25 mM Tris (pH 7.4), 1 mM EDTA, 50 mM KCl), heated to 90 °C, and slowly cool to room temperature.

Cell culture and oligonucleotide transfection. Cells were all purchased from ATCC (Manassas, VA). HeLa and MCF7 cells were cultured in Dulbecco's Modified Eagle's Medium (DMEM; Thermo Fisher Scientific) supplemented with 10 mM HEPES, 1 mM GlutaMax (Thermo Fisher), and 10% fetal bovine serum (FBS; Thermo Fisher). All cells were maintained at 37 °C in a humidified CO2 (5%) atmosphere. For oligonucleotides transfection, cells were plated at the concentration of 5000 cells/well (HeLa) or 10000 cells/well (MCF7) in 96-well plate one day prior to transfection. All oligonucleotides were transfected using Lipofectamine 3000 (Thermo Fisher Scientific) according to manufacturer's protocol. Transfection mixture was prepared by adding the desired amount of a concentrated oligonucleotide (10 μM) in a buffer containing 25 mM Tris (pH 7.4), 1 mM EDTA, and 50 mM KCl to a 5 μL solution containing 0.5 μL Lipofectamine 3000 and 4.5 μL Opti-MEM. The transfection mixture was then diluted to 30 μL in Opti-MEM. DMEM (70 μL) was added into each well followed by the 30 μL transfection mixture. Transfection was carried out at 37 °C in a humidified CO_2 (5%) atmosphere for 2 hours. After 2 hours, transfection mixture was removed and each well was washed with fresh DMEM twice and incubated in 100 μL DMEM for the duration of the experiment.

Toxicity assay. Various concentrations of oligonucleotide were transfected into cells as described above. The amount of Lipofectamine 3000 was kept constant among all samples in a given experiment. After 2 hours, the transfection mixture (in Opti-MEM) was removed from the cells and each well was washed with fresh DMEM twice and incubated in 100 μL DMEM. After 22 hours at 37°C, cell counting kit-8 (Sigma Aldrich) reagent was added and that the cells were incubated for 1 hour at 37°C. Cell viability was measured on a GloMax Discover multi-well plate reader from Promega Corp. (Madison, WI) by measuring absorbance at 450 nm. The background signal from a control well containing only the CCK-8 reagent in DMEM was subtracted from each sample and HeLa cells treated with Lipofectamine 3000 only were set as 100% viability. The half maximal effective concentration (EC_{50}) was determined using the equation:

$$Viability\ (\%) = \frac{100}{(1 + \frac{Concentration\ (nM)}{EC_{50}})}$$

Time-dependent toxicity assays were carried out and quantified similarly.

Cellular Uptake. Oligonucleotides were transfected into HeLa cells following protocol described above. After 2 hours, the media was removed and the cells were washed with 50 μL PBS twice and detached by 50 μL 0.25% trypsin at 37°C for 3 minutes. 50 μL DMEM supplemented with 10% FBS was then added to quench trypsin. Cellular uptake of the oligonucleotides was measured on an Accuri C6 Flow Plus Cytometer (BD Biosciences, San Jose, CA) using the FL4–APC filter (Ex: 640 nm; Em:BP 675/25 nm). All data was collected at a flow rate of 66 μL/min and has at least 10000 events collected.

Immunofluorescence. HeLa cells were plated on Ibidi 8-well chamber slides (ibidi GmbH, Gräfelfing, Germany) at the concentration of 3 x 10^4 cells/well. Oligonucleotides were transfected into HeLa cells following protocol mentioned above for 2 hours. All the following slide preparation were carried out Cells were washed with 5 x 150 μL PBS and fixed with freshly prepared 150 μL 4% formaldehyde for 30 minutes. Cells were washed with 3 x 50 μL PBS and permeabilized with 0.2% TritonX in PBS for 10 minutes. Cells were washed with 3 x 150 μL PBS and blocked with 1% BSA/ PBST (0.1% Tween-20 in PBS) for 30 minutes. Cells were washed with 3 x 150 μL PBST (3 minutes each) and stained with 1:100 primary antibody anti-fibrillarin (clone 38F3) (antibodies.com, Cambridge, United Kingdom) in 1% BSA/ PBST for 2 hours. Cells were washed with 3 x 150 μL PBST (3 minutes each) and stained with 1:500 secondary antibody Cy3-goat anti mouse IgG (Invitrogen; cat. No. A10521) in 1% BSA/ PBST for 1 hour. 1 μL of Hoechst 33342 was added to each well during the final 10 minutes of the secondary antibody incubation. After 10 minutes, cells were washed with 3 x 150 μL PBST and 3 x 150 μL PBS and soaked in 100 μL PBS. Confocal images were generated using Leica SP8 confocal microscope. Fluorescent and brightfield images were acquired using a HC PL APO 40x/1.10 W motCORR CS2 water immersion objective in conjunction with a 405 nm CW laser and a 470nm-670 nm white pulsed laser. PMT detector and hybrid detector HyD SMD were used for detection and all images were acquired using the Leica Application Suite X (version 5.0.2). All data analyses were conducted in ImageJ (v1.53q). All images were acquired under identical microscopy settings for a given experiment. Nucleus and nucleolus mean area fluorescence intensity were acquired using the freehand selection and measure feature in ImageJ.

Apoptosis assay. HeLa cells were transfected with 200 nM oligonucleotides following the method mentioned above. 10 μM camptothecin (CPT) treatment was used as a positive control. After 24 hours, cells were with washed with 50 μL PBS twice and detached by 50 μL 0.25% trypsin at 37°C for 3 minutes. 50 μL DMEM supplemented with 10% FBS was then added to quench

trypsin. Cells were then collected by centrifugation at 500 g for 5 minutes. The supernatant was removed and 500 μL Annexin V buffer (10 mM HEPES (pH7.4), 140 mM NaCl, 2.5 mM $CaCl_2$) was added to resuspend the cell pellet. 1 μL of AnnexinV-FITC reagent (Abcam) and 1 μL of propidium iodide (5 mg/mL) were added to the cell suspension and incubated at room temperature for 5 minutes. Fluorescence signal of cells was measured on an Accuri C6 Flow Plus Cytometer (BD Biosciences, San Jose, CA) using the FL1–FITC filter (Ex: 488 nm; Em:BP 533/30 nm) and the FL2–PE filter (Ex: 488 nm; Em:BP 585/40 nm). All data was collected at a flow rate of 66 μL/min and has at least 10000 events collected.

Total RNA isolation and high throughput sequencing. Total RNA was extracted from HeLa cell transfected with L-r$(GGAA)_8$, L-r$(GA)_{20}$, and L-r(GC/GC) negative control (lipofectamine only) using TRIzol reagent (Invitrogen, San Diego, CA) after 12 hour-oligonucleotide-treatment. All sample were conducted in biological triplicate. Total RNA was then purified using PureLink RNA mini kit (Invitrogen) and quantified using NanoDrop 2000c. 2 μg total RNA per sample were submitted to Azenta Life Science (South Plainfield, NJ) for high throughput sequencing to generate 150 bp paired-end reads. The RNA integrity analysis, cDNA library preparation, sequencing, and initial data quality check were performed by Azenta Life Science.

Bioinformatic analysis. Raw sequencing data quality was checked by FastQC (version 0.11.9) and trimmed by Trim Galore (version 0.6.6) before alignment. The clean reads of all 15 samples were aligned to human reference genome (hg38) using STAR (version 2.7.7a).[1] The reference human genome file and gene annotation file were both obtained from ENSEMBL. Both quality check and genome alignment were conducted using the high-performance research computing resources provided by Texas A&M University (http://hprc.tamu.edu) in the Linux operating system. The total reads for each transcript were quantified using FeatureCounts (Subread version 2.0.2).[2] Data visualization and differential gene expression of the RNA sequencing data was conducted on Rstudio (version 1.4.1717) with R version 4.1.0. Differential expression and statistical analysis were performed using DESeq2 (version 1.32.0).[3] Genes with adjusted p value <0.05 and $\log_2$ fold change (treated/ lipofectamine only) >1 or <-1 were considered as differential expressed.

To identify the pathway affected by L-oligonucleotides, Kyoto Encyclopedia of Genes and Genomes (KEGG) pathway analysis and Gene Ontology (GO) enrichment of differential expressed genes were carried out using clusterProfiler (version 4.0.5).[4] Identified KEGG pathways and GO terms with p value<0.05 were considered as significant enriched.

qRT-PCR analysis. Total RNA isolated from RNA-seq samples were reverse transcribed to cDNA using High-Capacity cDNA Reverse Transcription Kit (Applied Biosystems, Waltham, MA). Real-time quantitative PCR was carried out using TaqMan Fast Advanced Master Mix and TaqMan Gene Expression Assay (Applied Biosystems) according to manufacture protocol on a Bio-rad CFX96 optic module and C1000 thermocycler. The reaction was performed in triplicate and in a 10 μL-reaction consisting of sequence specific TaqMan primers for ACTB (Hs01060665_g1), CXCL1 (Hs00236937_m1), GADD45A (Hs00169255_m1), EGR1 (Hs00152928_m1), GDF15 (Hs00171132_m1), NFKB2 (Hs01028890_g1), TNF (Hs00174128_m1), TLR3 (Hs01551079_g1), TLR7 (Hs00152971_m1). C_T values of each sample were used to calculate ΔC_T and $\Delta\Delta C_T$ using ACTB as the internal reference. Relative gene expression was calculated using the $2^{-\Delta\Delta CT}$ method.

TLR siRNA knockdown

HeLa cells were plated at 30,000 cells/well in a 48-well plate one day prior to transfection. TLR was knock down by transfecting siRNA (TLR3, siRNA ID: s235; TLR7, siRNA ID: s27844) (Thermo Fisher Scientific) using Lipofectamine 3000 according to the manufacturer's protocol. After 24 hours, cells were detached and plated onto a 96 well-plate at 5,000 cells/well and incubate for another 24 hours prior to oligonucleotide transfection. The cells were then transfected with 50 nM of the indicated L-RNA as described above. After 22 hours, total RNA was extracted and analyzed by qRT-PCR.

Circular dichroism spectroscopy (CD). Oligonucleotides (10 μM) were folded in a buffer containing 25 mM Tris (pH 7.4), 1 mM EDTA, and either 50 mM KCl, 140 mM KCl, or 50 mM LiCl. To mimic transfection conditions, oligonucleotides were diluted into Opti-MEM supplemented with 25 mM Tris (pH 7.4), 1 mM EDTA, and 50 mM KCl. CD spectra were obtained from a 400 μL sample in quartz cuvette using an Applied Photophysics Chirascan spectrophotometer (Leatherhead, England) at 1 nm interval from 220 to 370 nm at room temperature.

Statistical Analysis

Statistical analysis was carried out on GraphPad Prism (v.9.3.1) and presented as means and standard deviations. Data sets within a given experiment were compared using unpaired one-way analysis of variance (ANOVA). Tukey's multiple comparisons test (α threshold = 0.05) was used for comparing significant differences between each condition tested.

Data Availability

Raw RNA-seq read and processed data were submitted to the NCBI GEO repository under the accession number GSE205338. The complete list of differentially expressed genes for L-r$(GGAA)_8$ and L-r$(GA)_{20}$ can be accessed in the Supplementary File 1 document.

S2. Supplementary Figures.

Figure S1

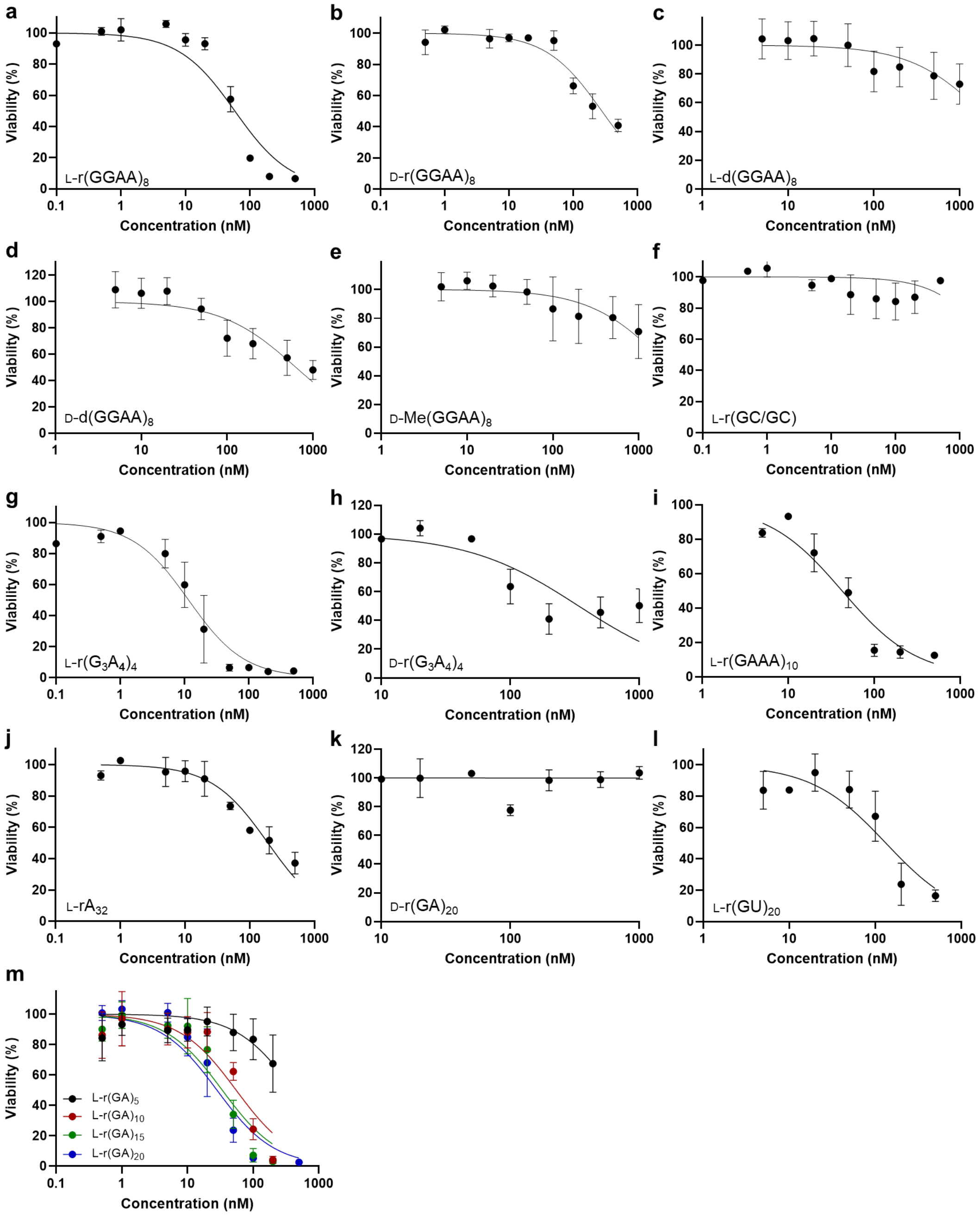

Figure S1. Dose response curves for L-r(GGAA)$_8$ (a), D-r(GGAA)$_8$ (b), L-d(GGAA)$_8$ (c), D-d(GGAA)$_8$ (d), D-Me(GGAA)$_8$ (e), L-r(GC/GC) (f), L-r(G$_3$A$_4$)$_4$ (g), D-r(G$_3$A$_4$)$_4$ (h), L-r(GAAA)$_{10}$ (i), L-rA$_{32}$ (j), D-r(GA)$_{20}$ (k), L-r(GU)$_{20}$ (l) and the L-r(GA)$_{20}$ series (m) based on CCK-8 assay. HeLa cells were transfected with indicated concentration of oligonucleotides for 2 hours and incubated in fresh DMEM for 46 hours as described in the methods section.

Figure S2

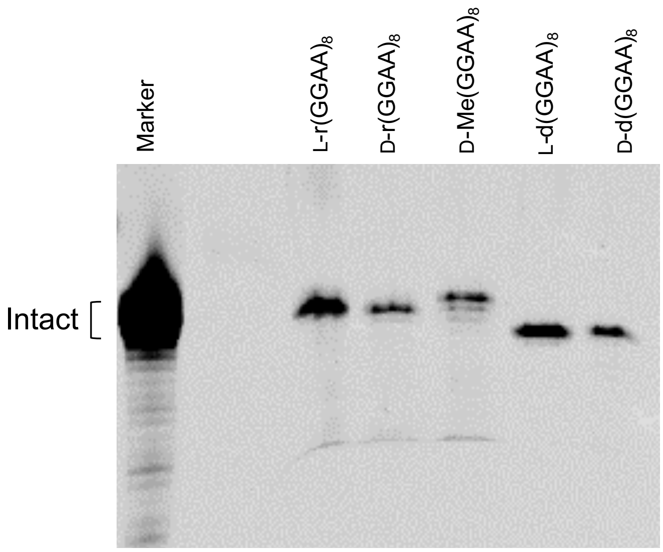

Figure S2*.* Denaturing PAGE (19:1 acrylamide:bisacrylamide) analysis of the indicated $(GGAA)_8$ variants extracted from HeLa cells after 24 hours. Marker: L-r$(GGAA)_8$.

Figure S3

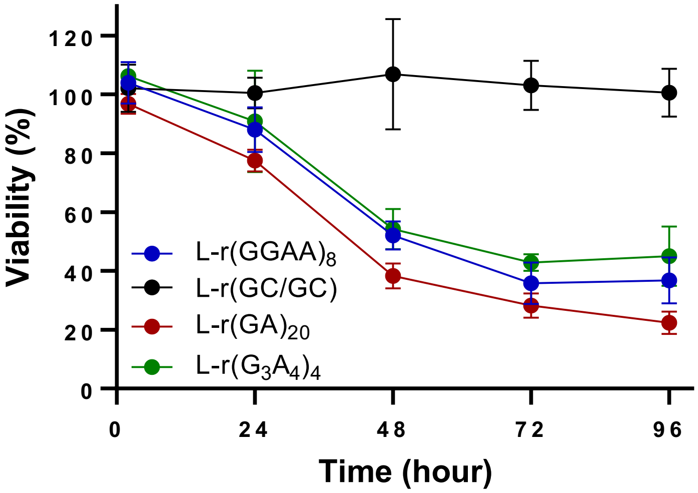

Figure S3. Time-dependent viability assay (CCK-8) of MCF7 cells treated with 200 nM of the indicated oligonucleotide. Data are mean ± S.D. (n = 3 biological replicates).

Figure S4.

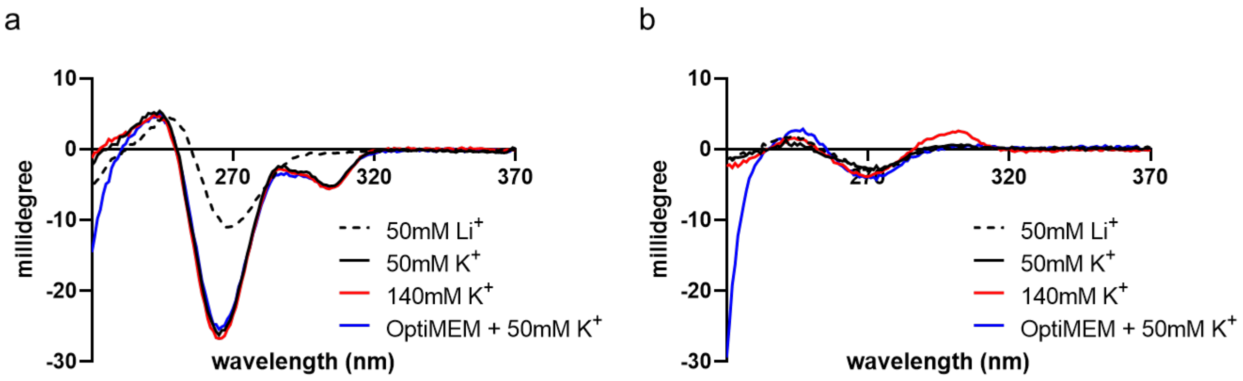

Figure S4. Circular dichroism (CD) spectra of L-r(GGAA)$_8$ (a) and L-r(GA)$_{20}$ (b) in buffer containing 25 mM Tris (pH 7.4), 1 mM EDTA, and either 50 mM KCl (50 mM K^+), 140 mM KCl (140 mM K^+), or 50 mM LiCl (50 mM Li^+). OptiMEM + 50 mM KCl indicates that the oligonucleotides were diluted into Opti-MEM supplemented with 25 mM Tris (pH 7.4), 1 mM EDTA, and 50 mM KCl to mimic transfection conditions. The CD spectrum of L-(GGAA)$_8$ (a) confirms the formation of a parallel G4 structure in the presence of K^+ as evident by a negative band at ~265 nm and a positive band at ~240 nm. Consistently, replacing K^+ with Li^+, which does not facilitate G-quadruplex formation, results in the loss of these features.

Figure S5

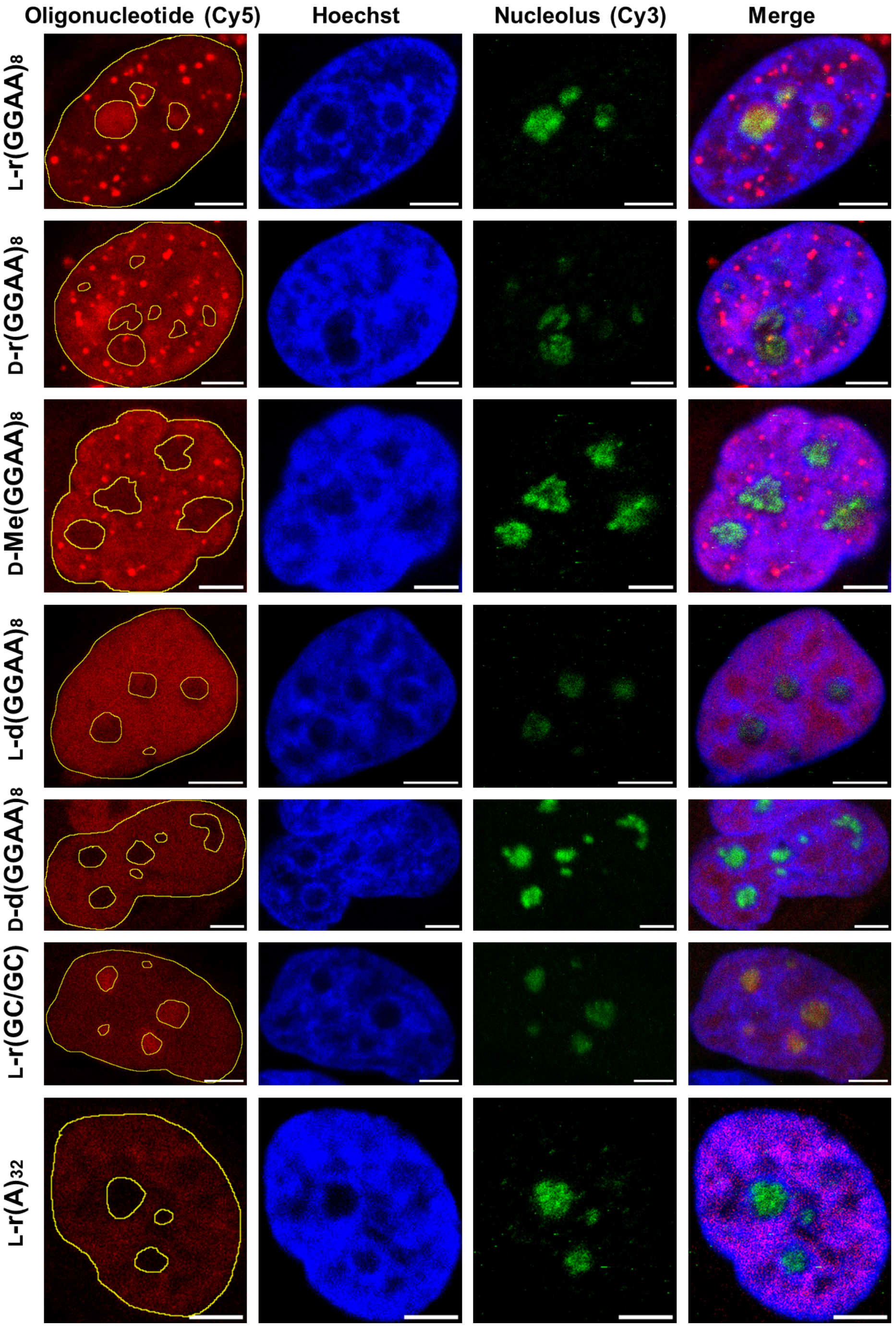

Figure S5. Representative fluorescent confocal microscopy images of HeLa cells transfected with 200 nM of the indicated oligonucleotides. Nucleolus was stained with an anti-fibrillarin antibody and a Cy3-labled secondary antibody. All scale bars: 5 µm. Yellow line in the Cy5 panel indicated the area used for nucleolus/nucleoplasm mean area fluorescence measurement.

Figure S6

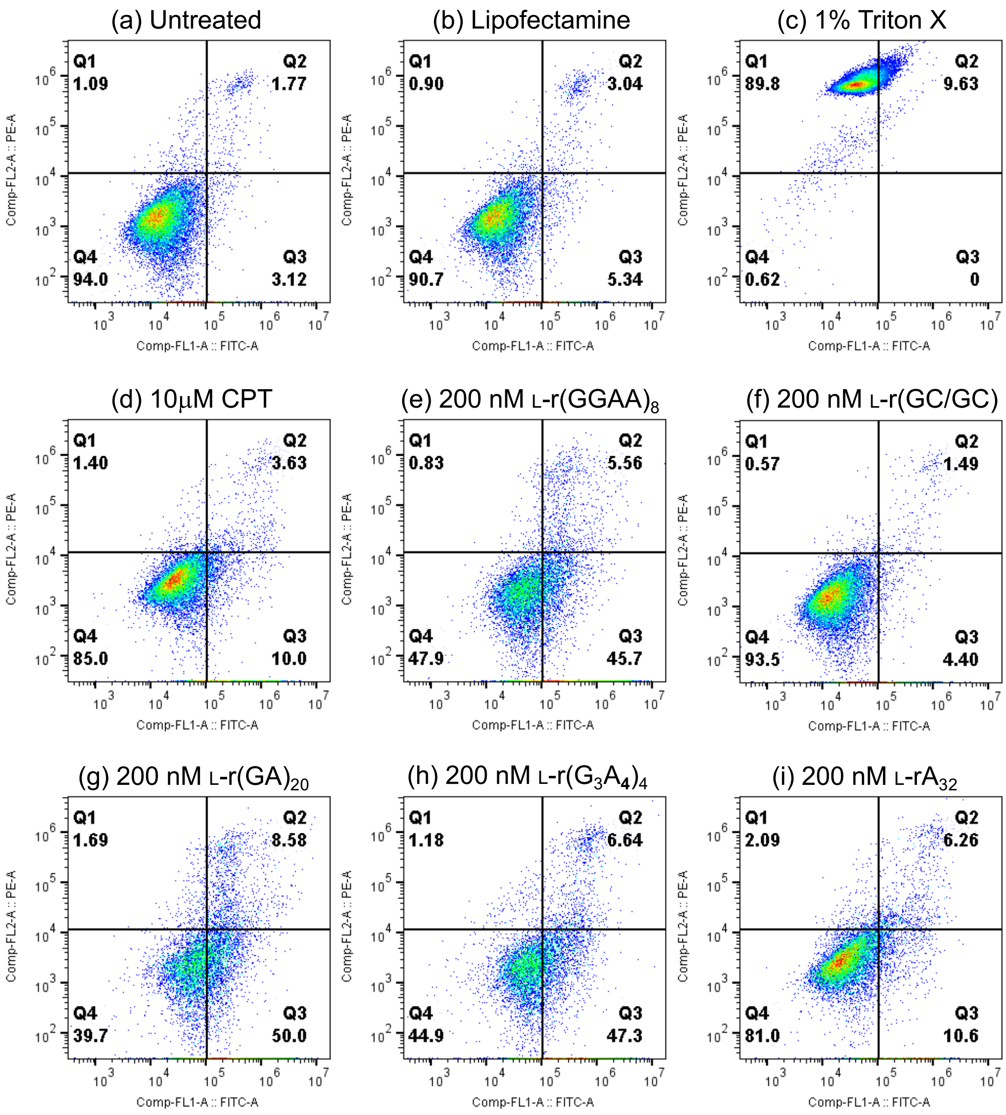

Legend

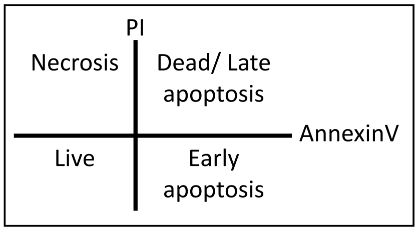

Figure S6. Representative quadrant dot plot of the results of flow cytometry after Annexin V and PI staining. HeLa cells were either untreated (a) or treated with lipofectamine only (b), 1% Triton X-100 (c), 10 µM CPT (d), 200 nM L-r(GGAA)$_8$ (e), 200 nM L-r(GC/GC) (f), 200 nM L-r(GA)$_{20}$ (g), 200 nM L-r(G$_3$A$_4$)$_4$ (h), or 200 nM L-rA$_{32}$ (i) for 24 hours. The x-axis represents Annexin V-FITC fluorescence intensity, while the y-axis represents propidium iodide fluorescence intensity. The cell population (%) in each quadrant is indicated.

Figure S7

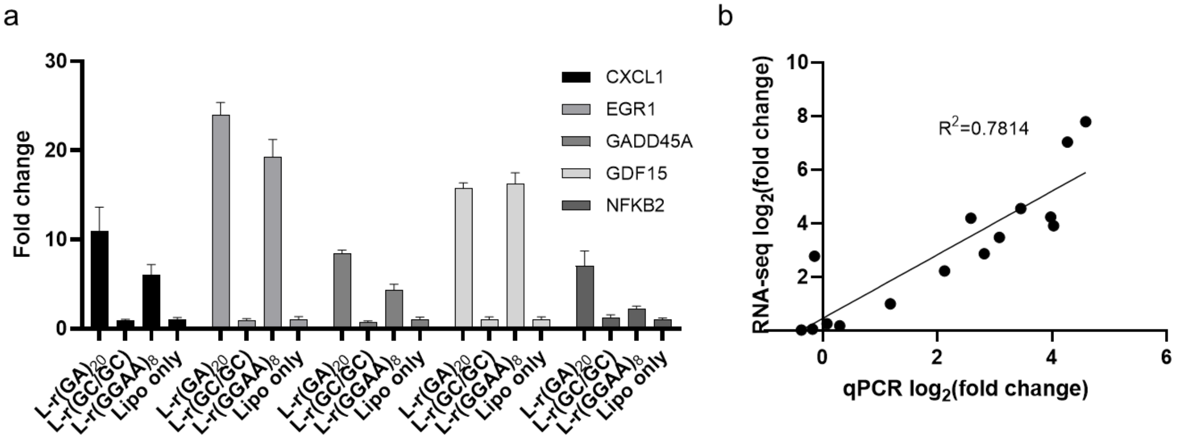

Figure S7. qRT-PCR verification of RNA-seq data. (a) Gene expression by qRT-PCR for CXCL1, EGR1, GADD45A, GDF15, and NFKB2 following 12-hour treatment with L-r$(GA)_{20}$, L-r(GC/GC) and L-r$(GGAA)_8$. Fold changes were calculated using the $2^{-\Delta\Delta Ct}$ method comparing the treated groups to the untreated control. Data are mean ± S.D. (n = 3 biological replicates). (b) Linear regression of the expression data from RNA sequencing and qRT-PCR.

Figure S8

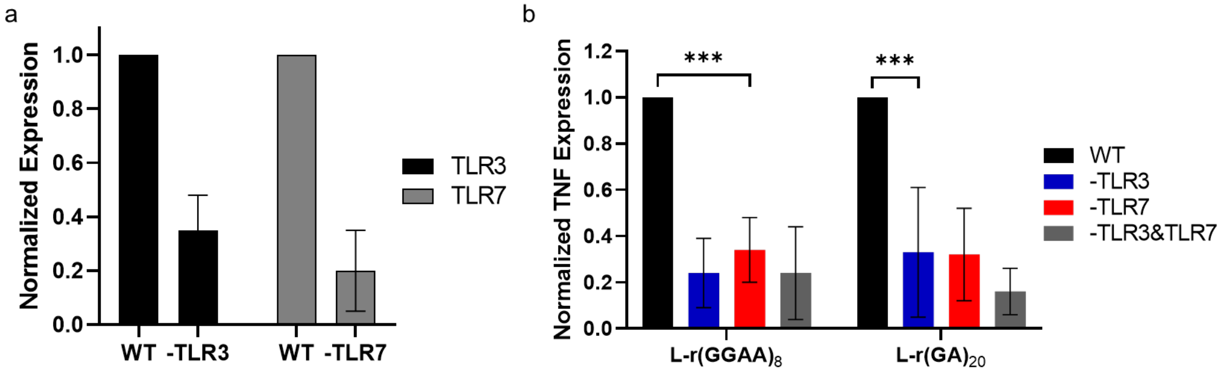

Figure S8. (a) qRT-PCR verification of siRNA-mediated knockdown of TLR3 and TLR7 after 72 hours. (b) TNF expression by qRT-PCR following 24-hour treatment of WT or TLR knockdown HeLa cells with 50 nM L-r$(GGAA)_8$ or L-r$(GA)_{20}$. Fold change was calculated using the $2^{-\Delta\Delta Ct}$ method comparing the treated groups to the untreated control. TNF expression was normalized to the wild type. Data are mean ± S.D. (n = 3 biological replicates). ****P* < 0.001.

Figure S9

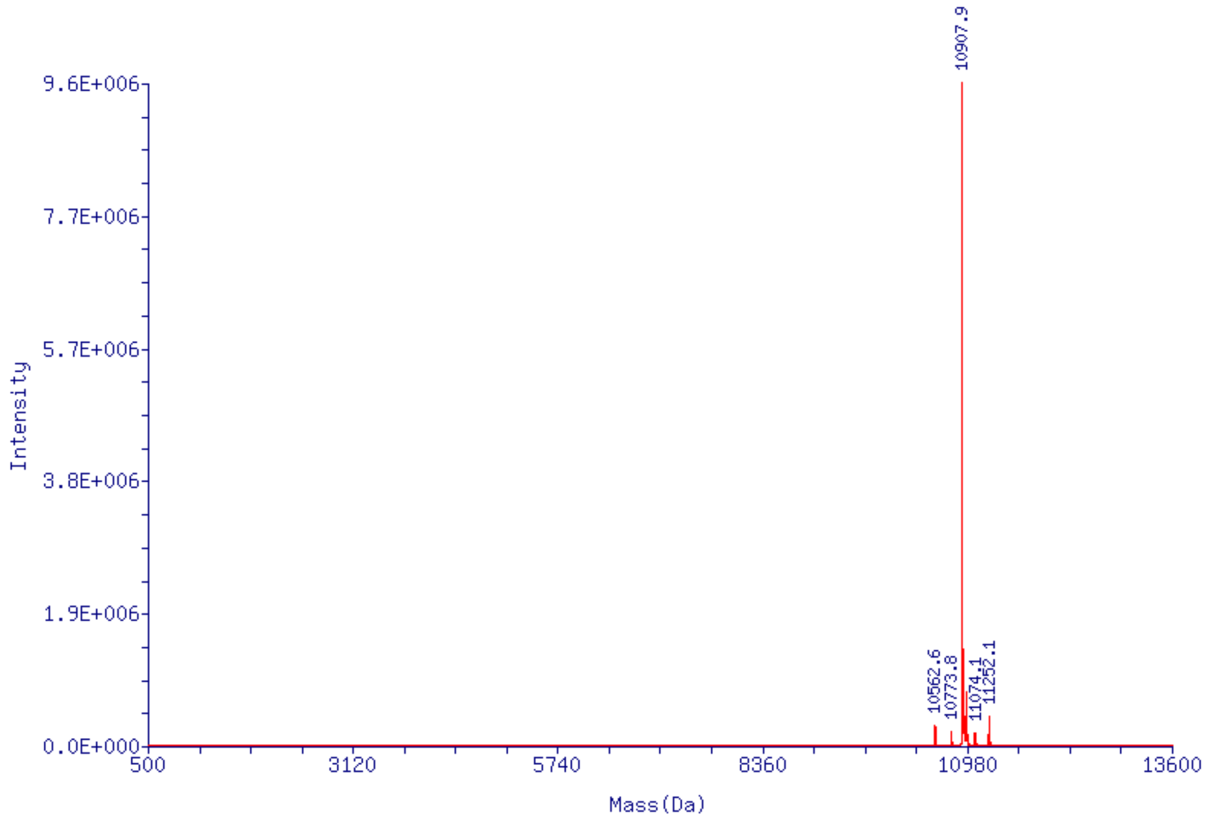

Figure S9. ESI-MS spectra of L-r(GGAA)$_8$ prepared by solid-phase synthesis. Mass calculated: 10907.9 Da; Mass found: 10907.9 Da.

Figure S10

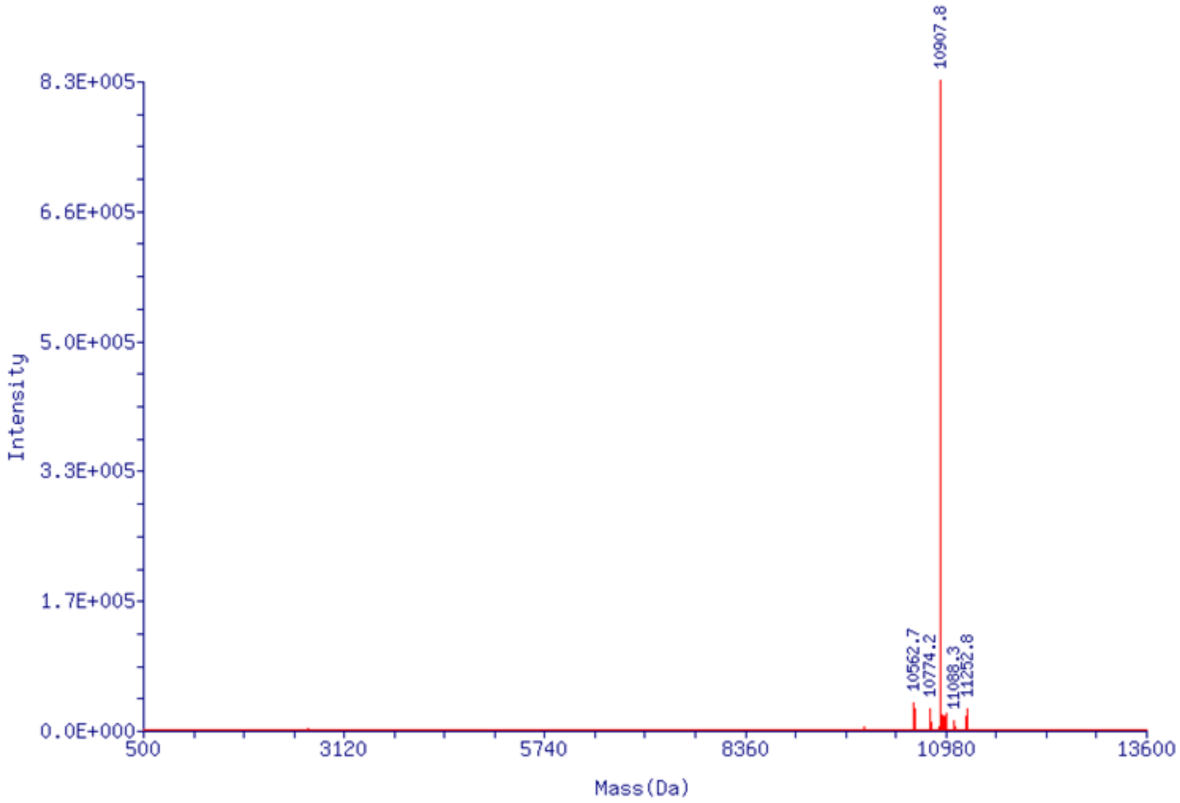

Figure S10. ESI-MS spectra of D-r(GGAA)$_8$ prepared by solid-phase synthesis. Mass calculated: 10907.9 Da; Mass found: 10907.8 Da.

Figure S11

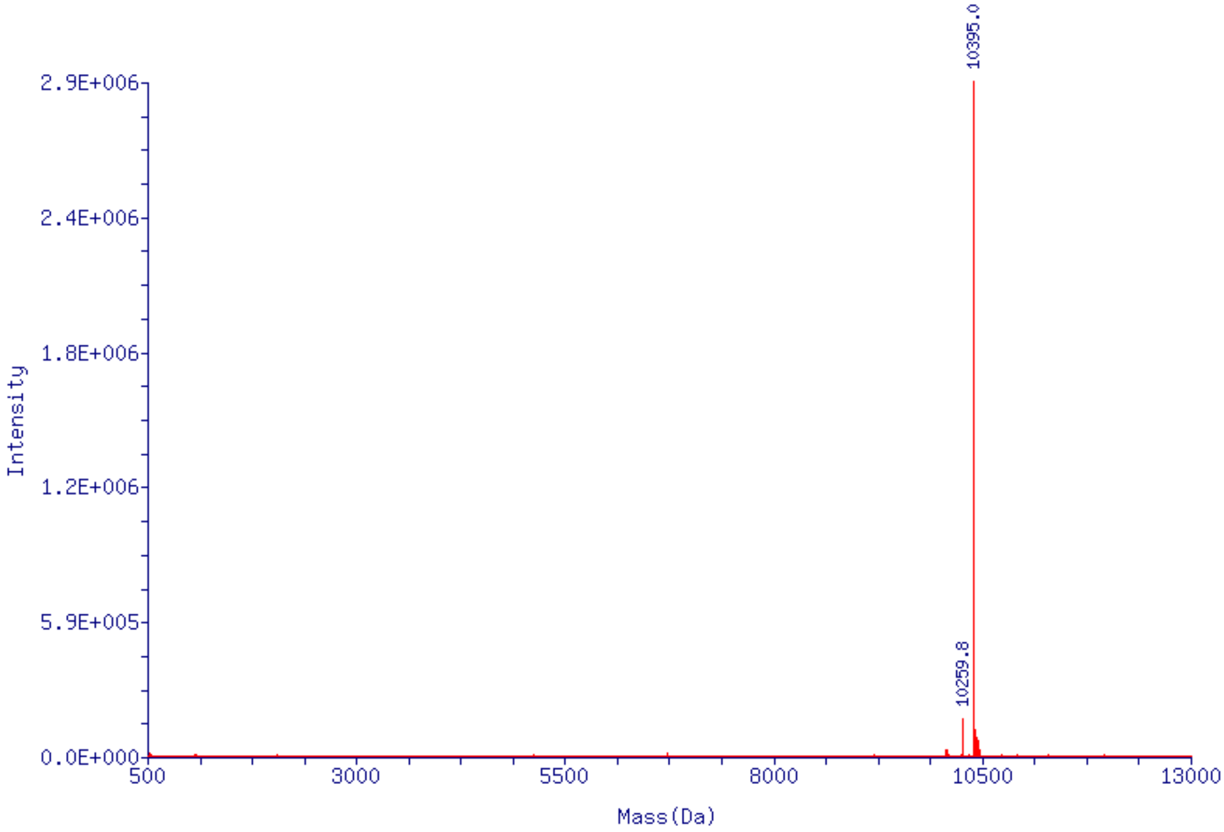

Figure S11. ESI-MS spectra of L-d(GGAA)$_8$ prepared by solid-phase synthesis. Mass calculated: 10395.9 Da; Mass found: 10395.0 Da.

Figure S12

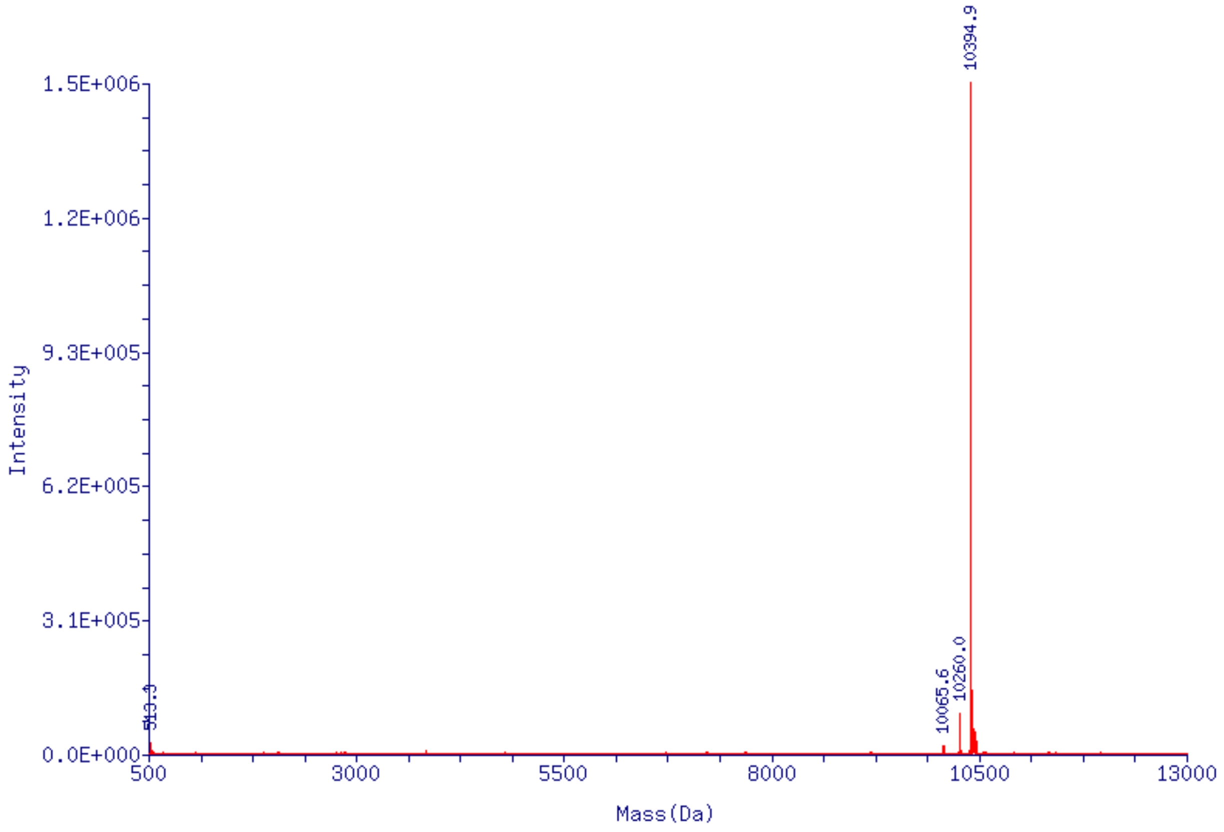

Figure S12. ESI-MS spectra of D-d(GGAA)$_8$ prepared by solid-phase synthesis. Mass calculated: 10395.9 Da; Mass found: 10394.9 Da.

Figure S13

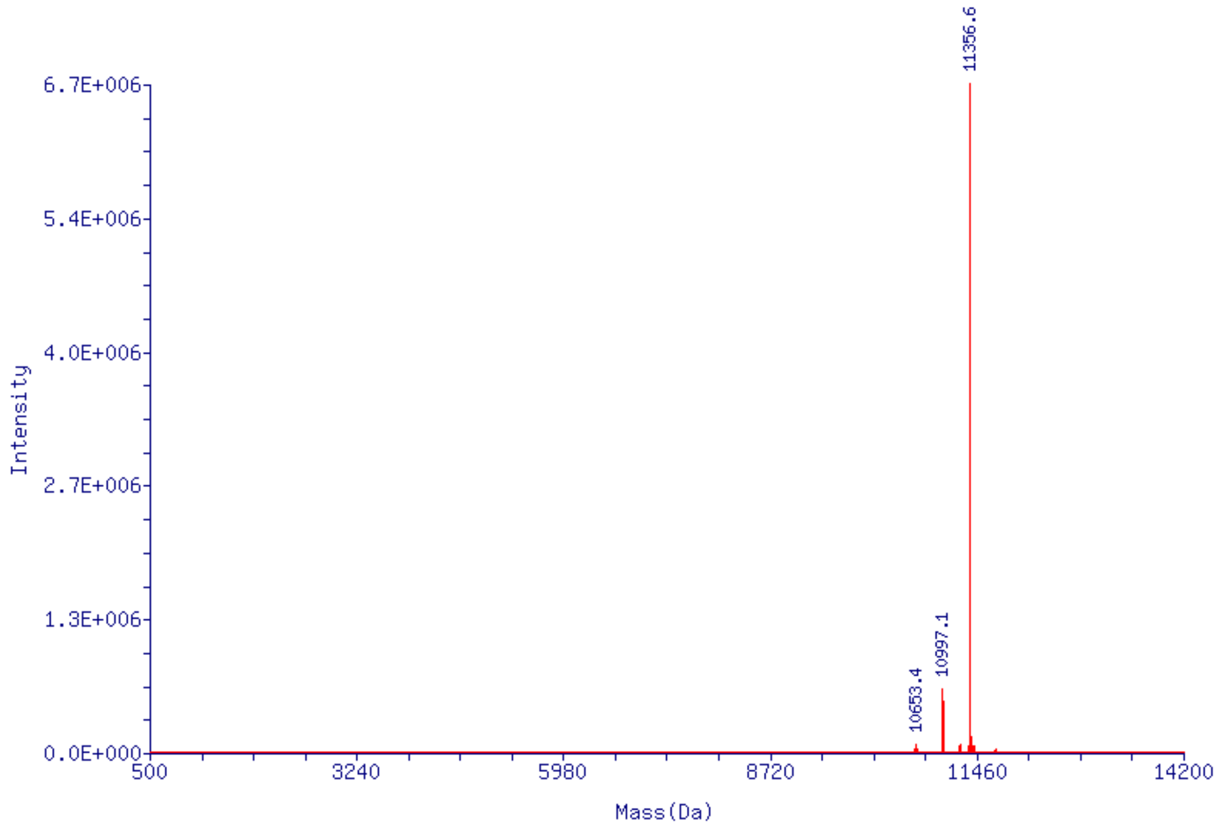

Figure S13. ESI-MS spectra of D-Me(GGAA)$_8$ prepared by solid-phase synthesis. Mass calculated: 11356.7 Da; Mass found: 11356.6 Da.

Figure S14

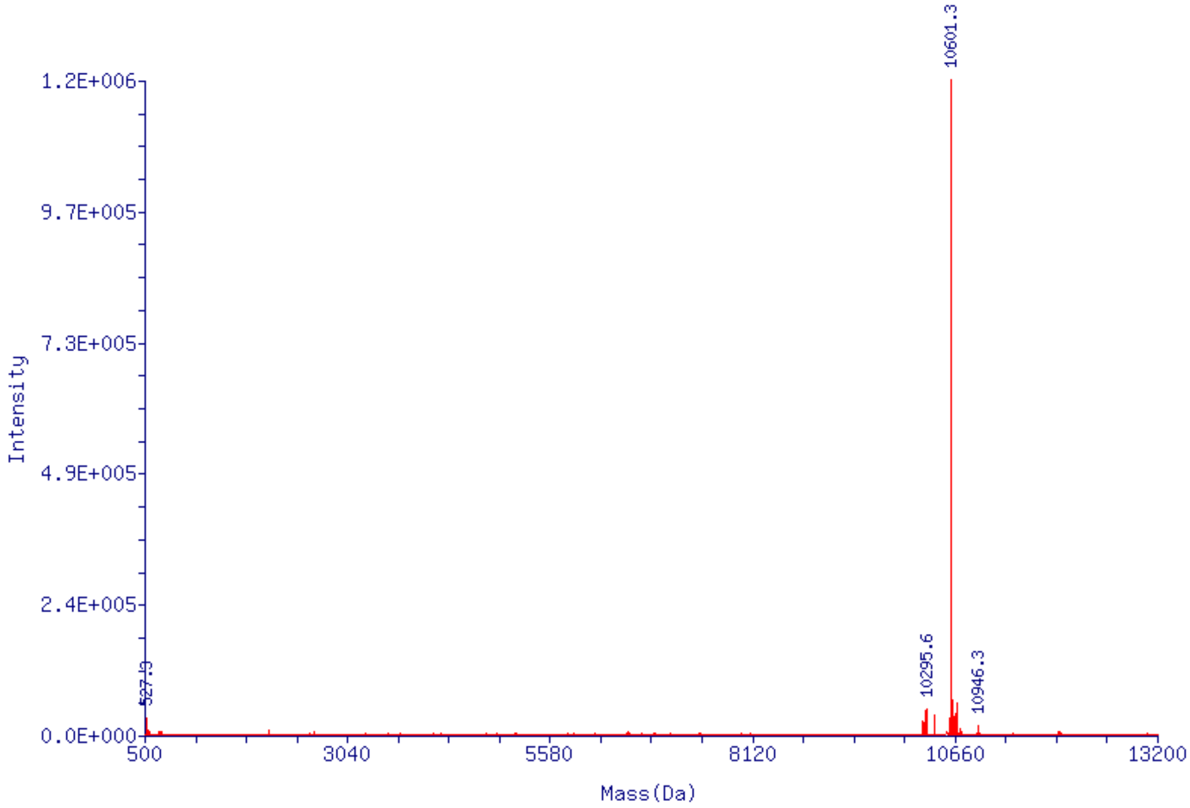

Figure S14. ESI-MS spectra of L-r(GC/GC) prepared by solid-phase synthesis. Mass calculated: 10601.5 Da; Mass found: 10601.3 Da.

Figure S15

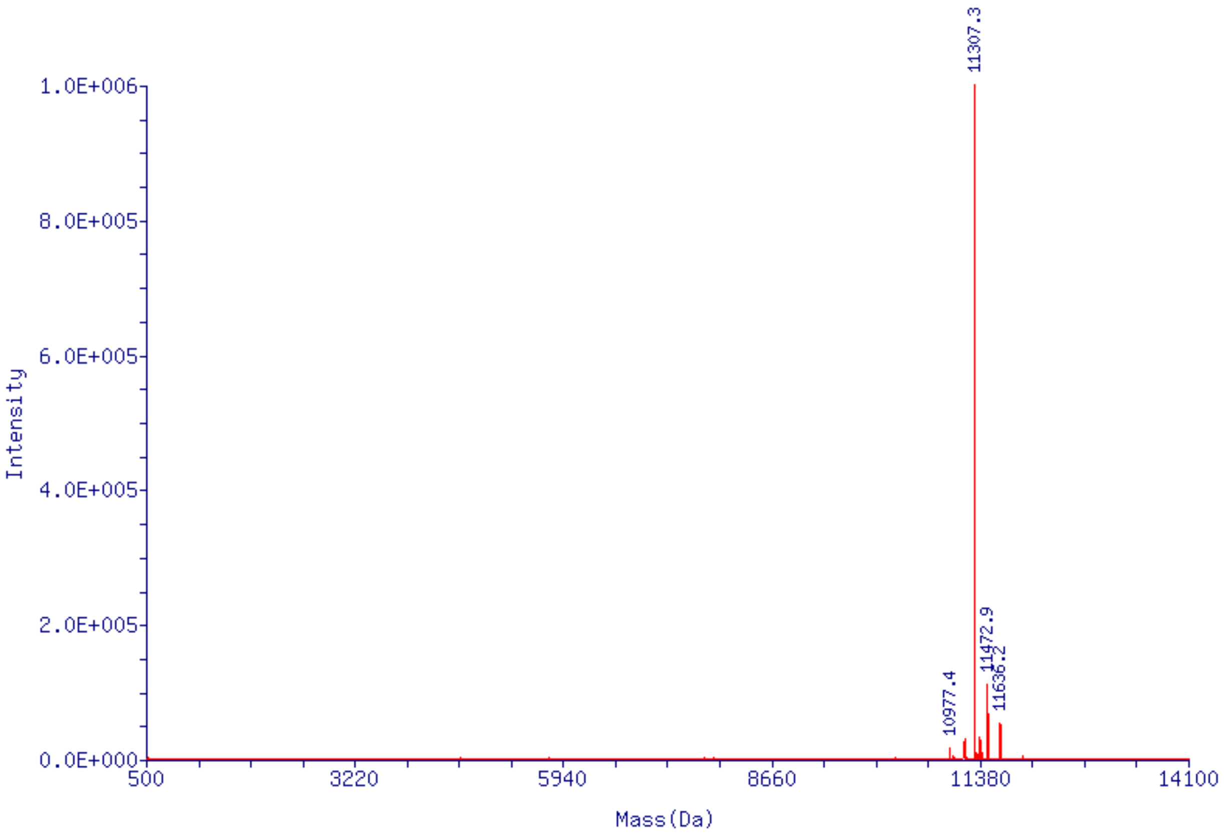

Figure S15. ESI-MS spectra of L-rA_{32} prepared by solid-phase synthesis. Mass calculated: 11305.7 Da; Mass found: 11307.3 Da.

Figure S16

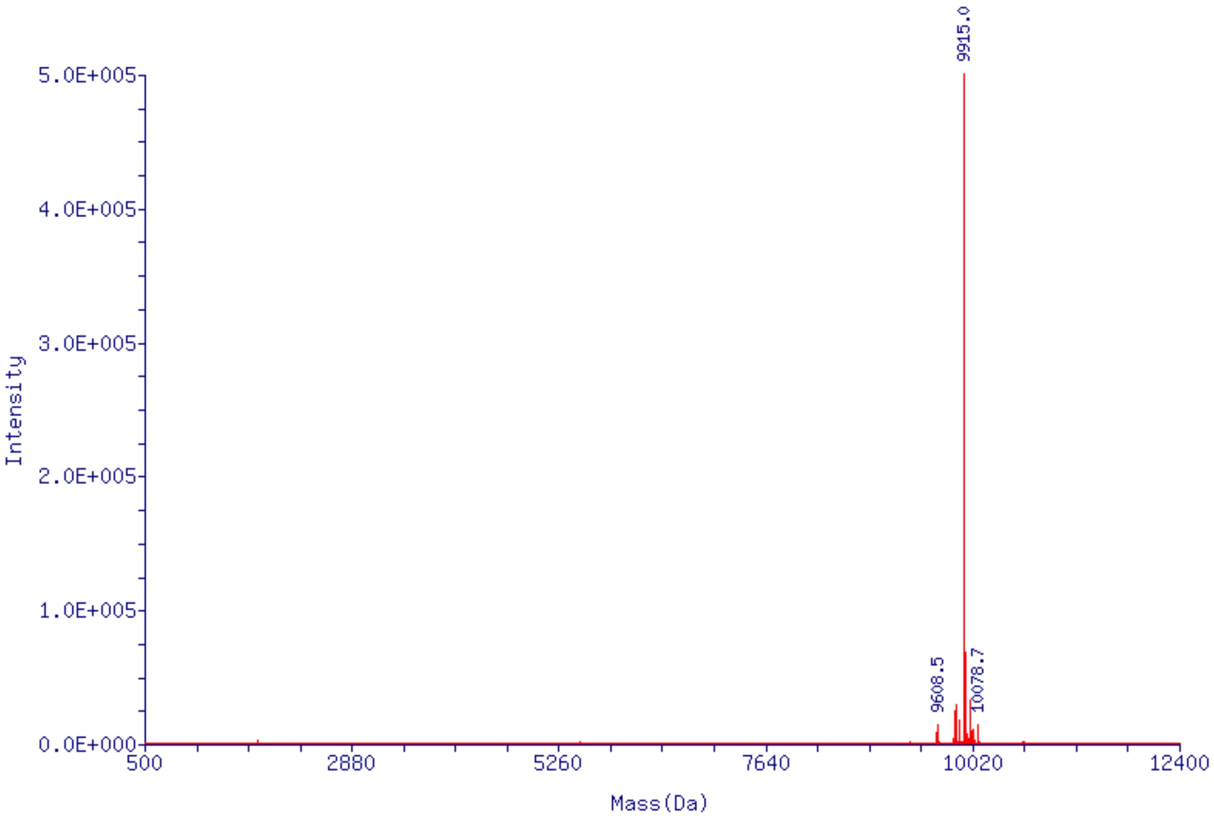

Figure S16. ESI-MS spectra of L-rU$_{32}$ prepared by solid-phase synthesis. Mass calculated: 9914.6 Da; Mass found: 9915.0 Da.

Figure S17

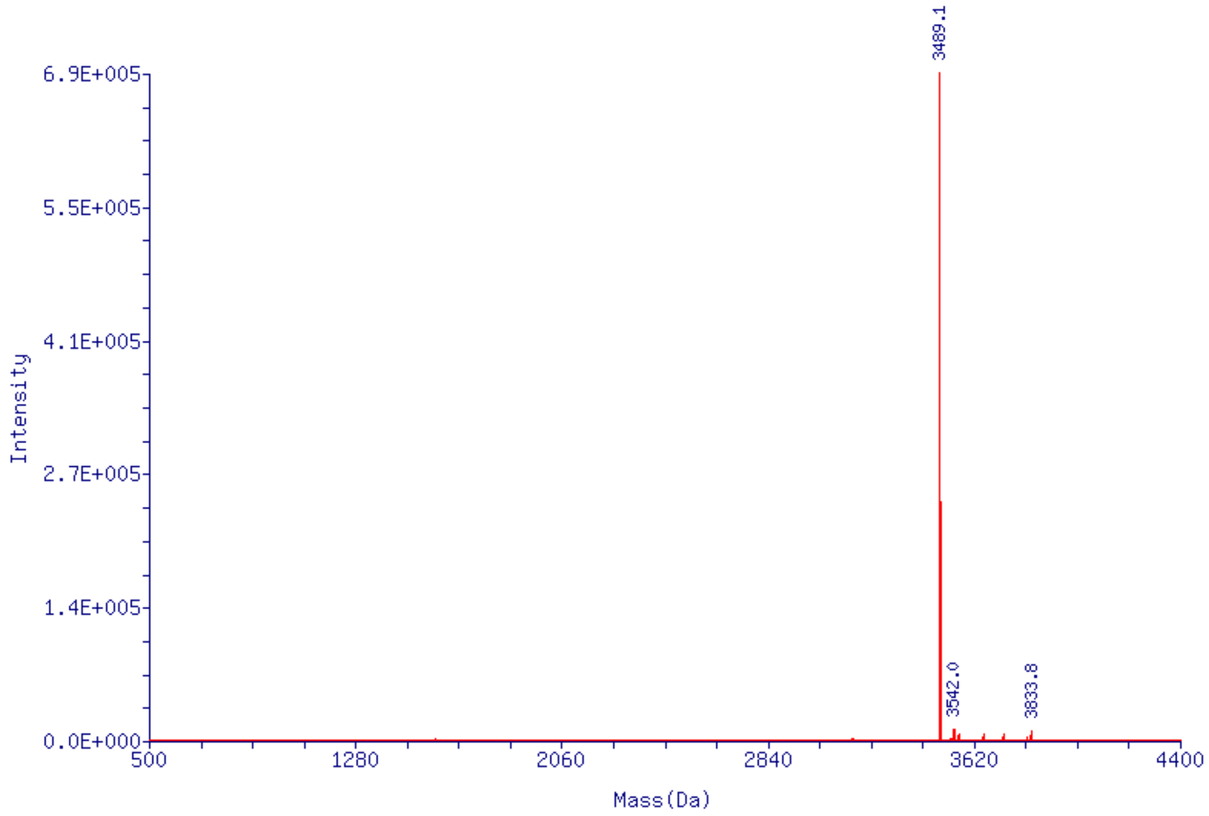

Figure S17. ESI-MS spectra of L-r(GA)$_5$ prepared by solid-phase synthesis. Mass calculated: 3489.3 Da; Mass found: 3489.1 Da.

Figure S18

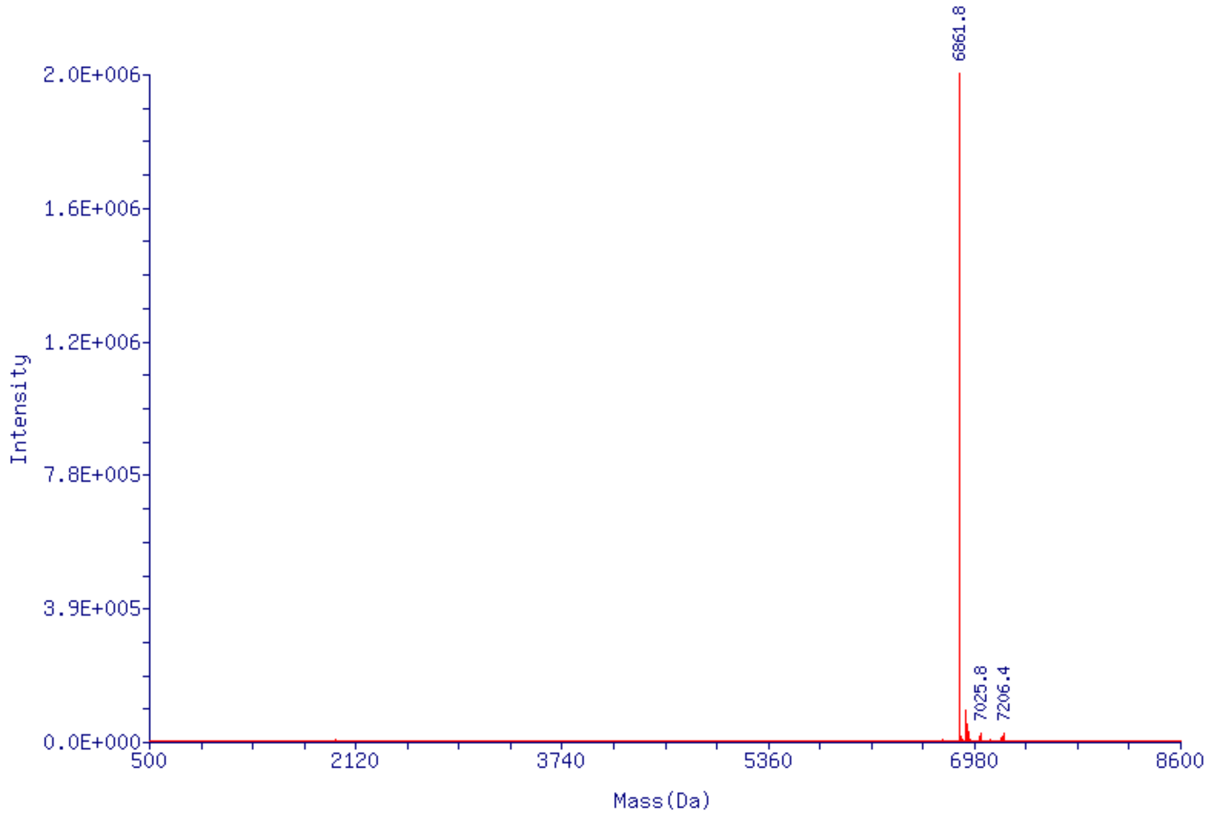

Figure S18. ESI-MS spectra of L-r(GA)$_{10}$ prepared by solid-phase synthesis. Mass calculated: 6861.4 Da; Mass found: 6861.8 Da.

Figure S19

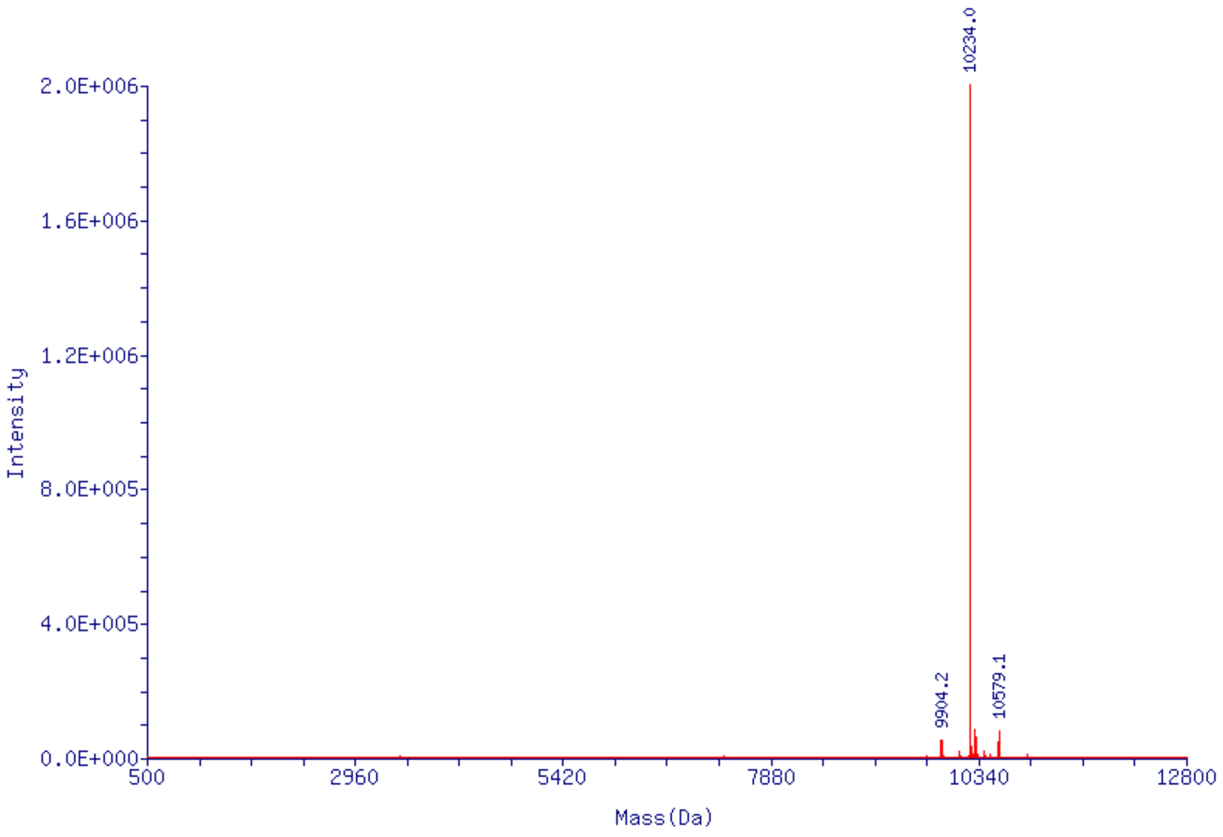

Figure S19. ESI-MS spectra of L-r(GA)$_{15}$ prepared by solid-phase synthesis. Mass calculated: 10233.5 Da; Mass found: 10234.0 Da.

Figure S20

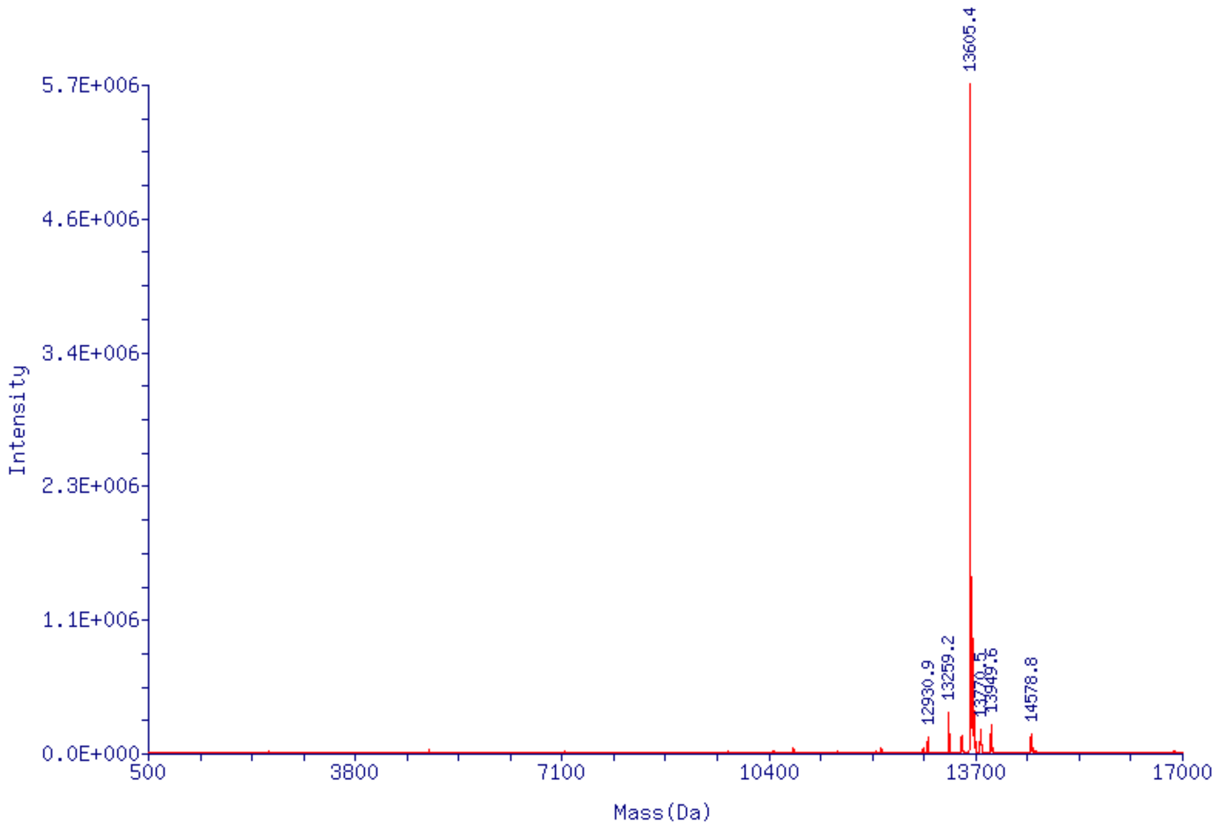

Figure S20. ESI-MS spectra of L-r(GA)$_{20}$ prepared by solid-phase synthesis. Mass calculated: 13605.5 Da; Mass found: 13605.4 Da.

Figure S21

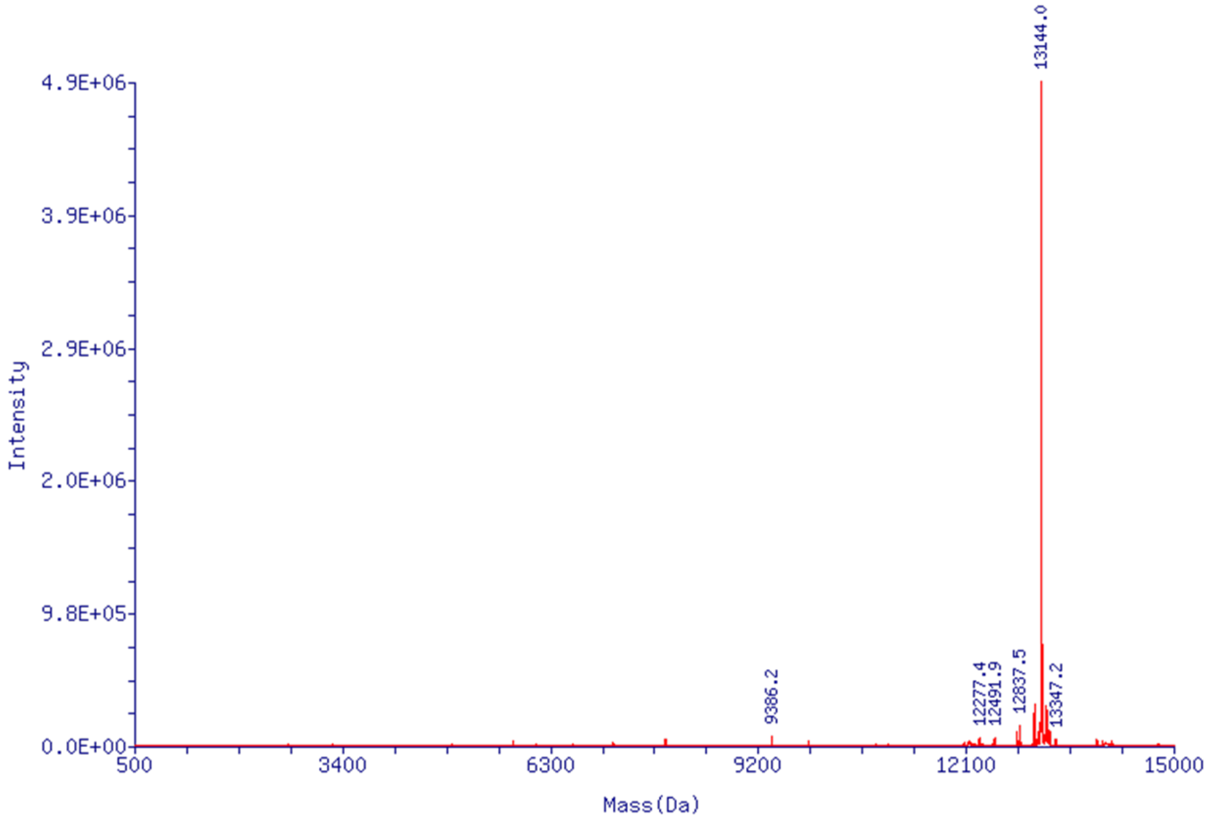

Figure S21. ESI-MS spectra of L-r(GU)$_{20}$ prepared by solid-phase synthesis. Mass calculated: 13144.7 Da; Mass found: 13144.0 Da.

Figure S22

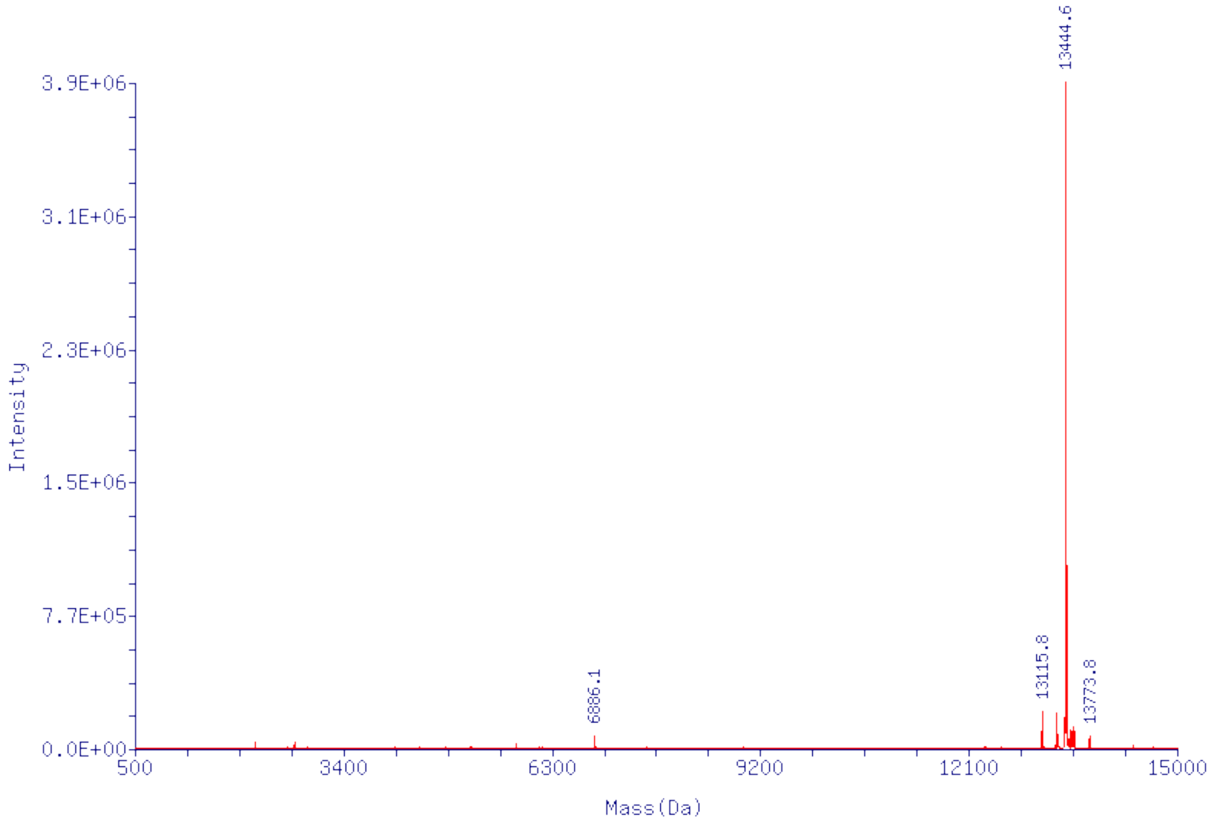

Figure S22. ESI-MS spectra of L-r(GAAA)$_{10}$ prepared by solid-phase synthesis. Mass calculated: 13445.5 Da; Mass found: 13444.6 Da.

Figure S23

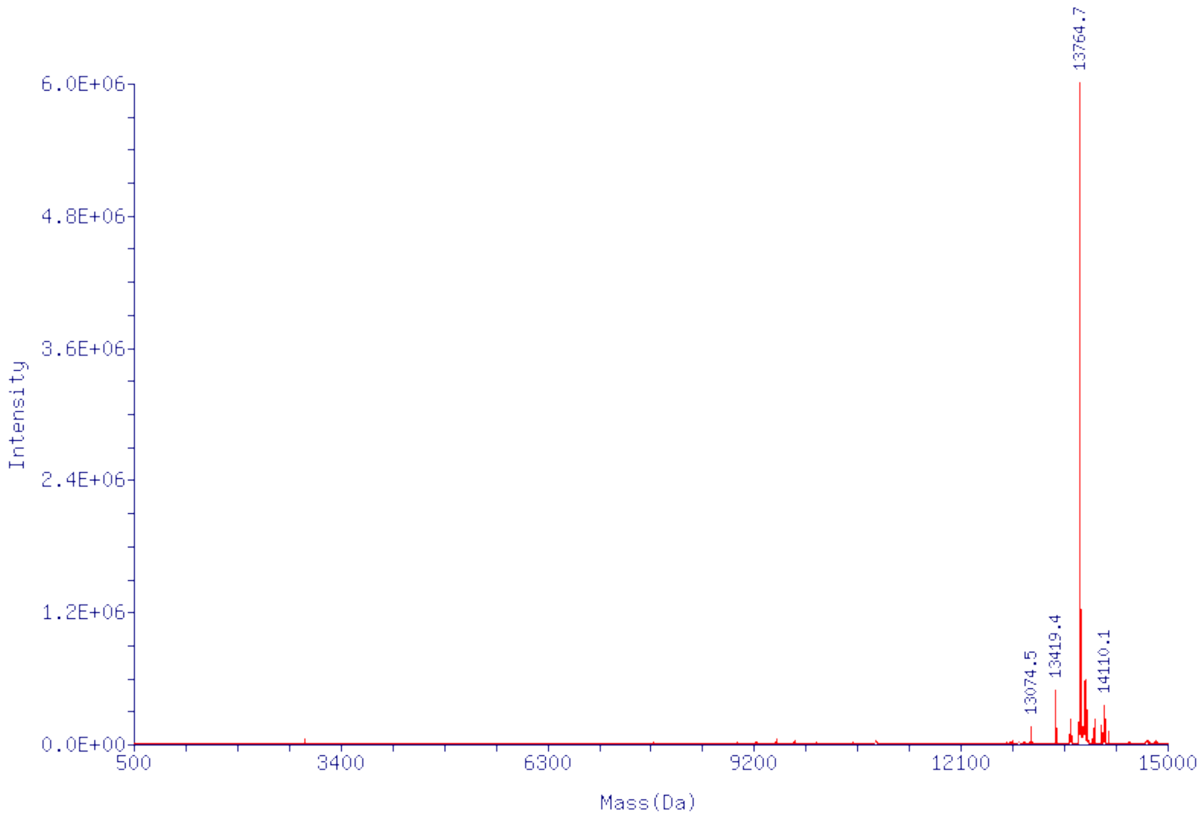

Figure S23. ESI-MS spectra of L-r(GGGA)$_{10}$ prepared by solid-phase synthesis. Mass calculated: 13765.5 Da; Mass found: 13764.7 Da.

S3. Supplementary Tables.

Table S1. Names and sequences of oligonucleotides used in this work. L-oligonucleotide (blue) and D-oligonucleotide (black) are indicated by color. /Cy5/ = cyanine5 dye; 2'-OMethyl nucleotides are indicated as underlined.

Sequence Name	Sequence Identity 5′→3′	DNA/RNA
L-r(GGAA)$_8$	GGAAGGAAGGAAGGAAGGAAGGAAGGAAGGAA-/Cy5/	RNA
D-r(GGAA)$_8$	GGAAGGAAGGAAGGAAGGAAGGAAGGAAGGAA-/Cy5/	RNA
L-d(GGAA)$_8$	GGAAGGAAGGAAGGAAGGAAGGAAGGAAGGAA-/Cy5/	DNA
D-d(GGAA)$_8$	GGAAGGAAGGAAGGAAGGAAGGAAGGAAGGAA-/Cy5/	DNA
D-Me(GGAA)$_8$	GGAAGGAAGGAAGGAAGGAAGGAAGGAAGGAA-/Cy5/	RNA
L-r(GC/GC)	GCGCGCGCGCGCGCGAGAGCGCGCGCGCGCGC-/Cy5/	RNA
L-rA$_{32}$	AAAAAAAAAAAAAAAAAAAAAAAAAAAAAAAA-/Cy5/	RNA
L-rU$_{32}$	UUUUUUUUUUUUUUUUUUUUUUUUUUUUUUUU-/Cy5/	RNA
L-r(GA)$_5$	GAGAGAGAGA-/Cy5/	RNA
L-r(GA)$_{10}$	GAGAGAGAGAGAGAGAGAGA-/Cy5/	RNA
L-r(GA)$_{15}$	GAGAGAGAGAGAGAGAGAGAGAGAGAGAGA-/Cy5/	RNA
L-r(GA)$_{20}$	GAGAGAGAGAGAGAGAGAGAGAGAGAGAGAGAGAGA GAGA-/Cy5/	RNA
D-r(GA)$_{20}$	GAGAGAGAGAGAGAGAGAGAGAGAGAGAGAGAGAGA GAGA	RNA
L-r(G$_3$A$_4$)$_4$	GGGAAAAGGGAAAAGGGAAAAGGGAAAA-/Cy5/	RNA
D-r(G$_3$A$_4$)$_4$	GGGAAAAGGGAAAAGGGAAAAGGGAAAA	RNA
L-r(GAAA)$_{10}$	GAAAGAAAGAAAGAAAGAAAGAAAGAAAGAAAGAAAGA AA-/Cy5/	RNA
L-r(GGGA)$_{10}$	GGGAGGGAGGGAGGGAGGGAGGGAGGGAGGGAGGG AGGGA-/Cy5/	RNA
L-r(GU)$_{20}$	GUGUGUGUGUGUGUGUGUGUGUGUGUGUGUGUGUG UGUGU-/Cy5/	RNA

Table S2. Overexpression of the pro-inflammatory cytokines and pro-apoptotic genes. Refer to Supplementary File 1. ns = not significant (adjusted P>0.05).

Cytokines	Fold of upregulation	
	L-r(GGAA)$_8$	L-r(GA)$_{20}$
TNF	ns	39.5
IL-1α	5.7	16.9
IL-6	4.5	7.9
IL-12	10.8	ns
CXCL1	130.8	174.7
CXCL2	5.8	12.2
CXCL8	9.7	20.9
Pro-apoptotic genes	L-r(GGAA)$_8$	L-r(GA)$_{20}$
GADD153	2.5	3.8
Bak	1.6	2.3
TNFRSF10B	3.0	3.4
Fas	2.9	2.3
BBC3	3.0	2.0
PMAIP1	2.3	2.7

S4. References.

1. Dobin, A.; Davis, C. A.; Schlesinger, F.; Drenkow, J.; Zaleski, C.; Jha, S.; Batut, P.; Chaisson, M.; Gingeras, T. R., STAR: ultrafast universal RNA-seq aligner. *Bioinformatics* **2013,** *29*, 15-21.
2. Liao, Y.; Smyth, G. K.; Shi, W., The R package Rsubread is easier, faster, cheaper and better for alignment and quantification of RNA sequencing reads. *Nucleic Acids Res.* **2019,** *47*, e47.
3. Love, M. I.; Huber, W.; Anders, S., Moderated estimation of fold change and dispersion for RNA-seq data with DESeq2. *Genome. Biol.* **2014,** *15*, 550.
4. Wu, T.; Hu, E.; Xu, S.; Chen, M.; Guo, P.; Dai, Z.; Feng, T.; Zhou, L.; Tang, W.; Zhan, L.; Fu, X.; Liu, S.; Bo, X.; Yu, G., clusterProfiler 4.0: A universal enrichment tool for interpreting omics data. *Innovation (N Y)* **2021,** *2*, 100141.
